# Supplementary material for: The lupus susceptibility allele DRB1*03:01 encodes a disease-driving epitope
Source: Commun Biol. 2022 Jul 28;5:751. doi: 10.1038/s42003-022-03717-x (PMC9334592; doi:10.1038/s42003-022-03717-x)
Supplement: Supplementary file 1 — Supplementary Information [file 42003_2022_3717_MOESM1_ESM.pdf]

Supplemental Material

The lupus susceptibility allele *DRB1*03:01* encodes a disease-driving epitope

Bruna Miglioranza Scavuzzi[1,6], Vincent van Drongelen[1,6], Bhavneet Kaur[1], Jennifer Callahan Fox[1], Jianhua Liu[1], Raquel A. Mesquita-Ferrari[1], J. Michelle Kahlenberg[1], Evan A. Farkash[2], Fernando Benavides[3], Frederick W. Miller[4], Amr H. Sawalha [1,5], Joseph Holoshitz[1,*]

[1] Department of Internal Medicine, University of Michigan, Ann Arbor, MI, 48109, USA.

[2] Department of Pathology, University of Michigan, Ann Arbor, MI, 48109, USA.

[3] Department of Epigenetics and Molecular Carcinogenesis, MD Anderson Cancer Center, Houston, TX, 77030, USA.

[4] Environmental Autoimmunity Group, National Institute of Environmental Health Sciences, Research Triangle Park, NC, 27709, USA.

[5] Departments of Pediatrics and Internal Medicine, University of Pittsburgh, Pittsburgh, PA 15224, USA.

[6] These authors contributed equally.

*Correspondence: jholo@umich.edu (J.H.)

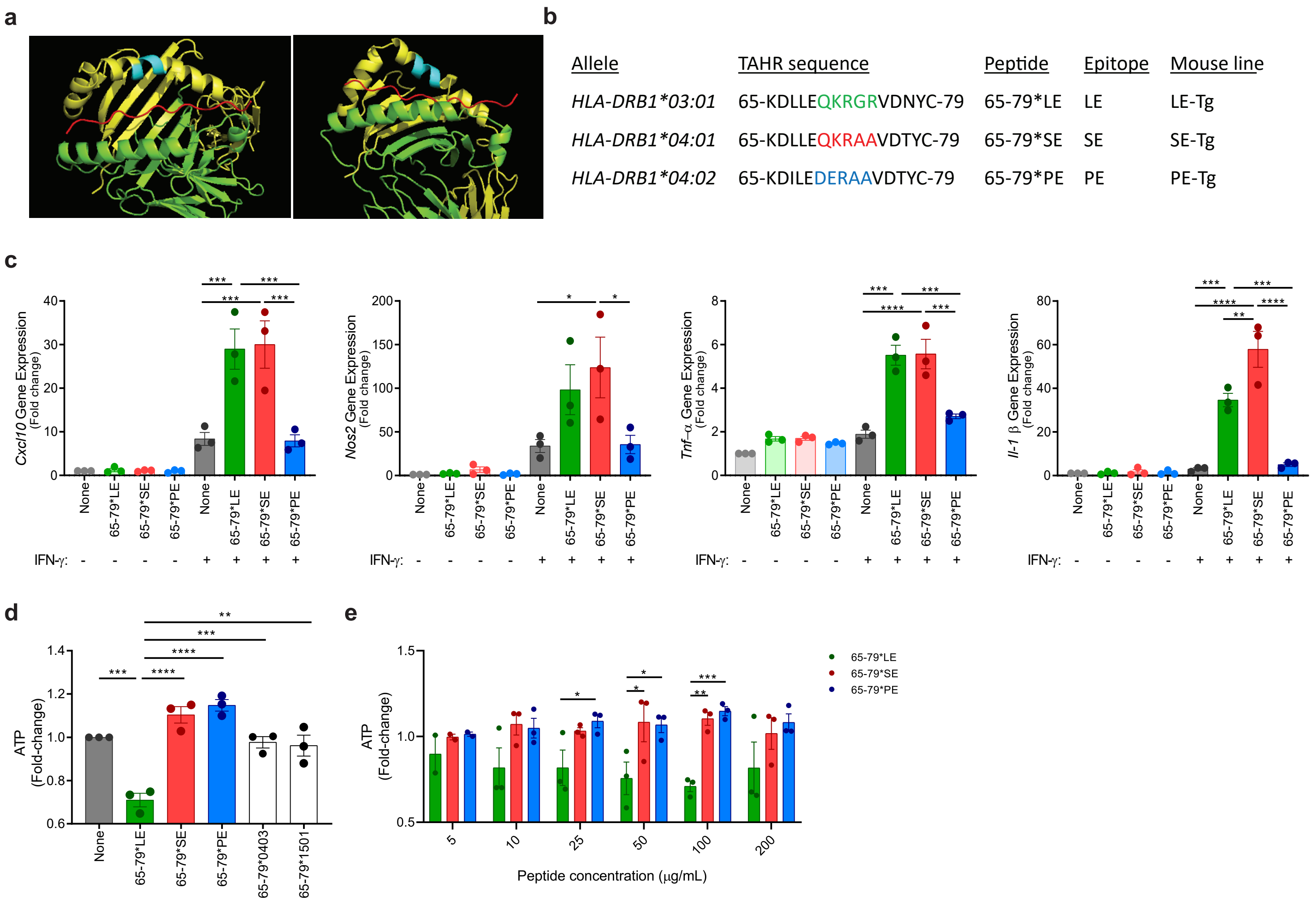

Supplementary Fig. 1: Background information and terminologies. Related to Figs. 1, 2, 3 and 7.

a, 'Top' (left) and 'side' (right) views of a three-dimensional ribbon model of the HLA-DR3 molecule focusing on the cusp region. Green: DRα chain; Yellow: DRβ chain; Red: groove peptide (CLIP). The TAHR polymorphic residues 70-74 are highlighted in cyan blue. Images are based on the known crystal structure of DR3 with the CLIP peptide (1A6A.pdb).
b, A list of the *DRB1* alleles studied here along with their respective coded TAHR 65-79 amino acid sequences, designations in this study of the different 15mer synthetic peptides corresponding to these TAHRs, short functional designation of allelic epitopes, and *DRB1* allele-specific transgenic mouse lines used in this study.
c, IFN-γ is an obligatory co-factor for epitope-activated transcriptional modulation. qRT-PCR analyses of marker genes in RAW 264.7 macrophages treated with or without 100 µg/mL 65-79*LE (green), 65-79*SE (red) or 65-79*PE (blue) for 72 hours, in the presence (solid-color bars) or absence (light-color bars) of IFN-γ (5 ng/mL).
d, Allele specificity. Intracellular ATP levels in RAW 264.7 macrophages exposed to epitope-specific 15mer peptides studied here: 65-79*LE (green), 65-79*SE (red), 65-79*PE (blue), along with two additional control synthetic 15mer allelic peptides: 65-79*0403 (corresponding to allele *DRB1*04:03*) and 65-79*1501 (corresponding to allele *DRB1*15:01*) in the presence of IFN-γ.
e, Intracellular ATP levels in RAW 264.7 macrophages treated with various doses of 65-79*LE, 65-79*SE and 65-79*PE and IFN-γ (5 ng/mL).
Data (**c**-**e**) represent mean ± SEM of 3 independent experiments. Two-way (**c**,**e**) or One-way (**d**) ANOVA, * $p < 0.05$, ** $p < 0.01$, *** $p < 0.001$, ****$p < 0.0001$.

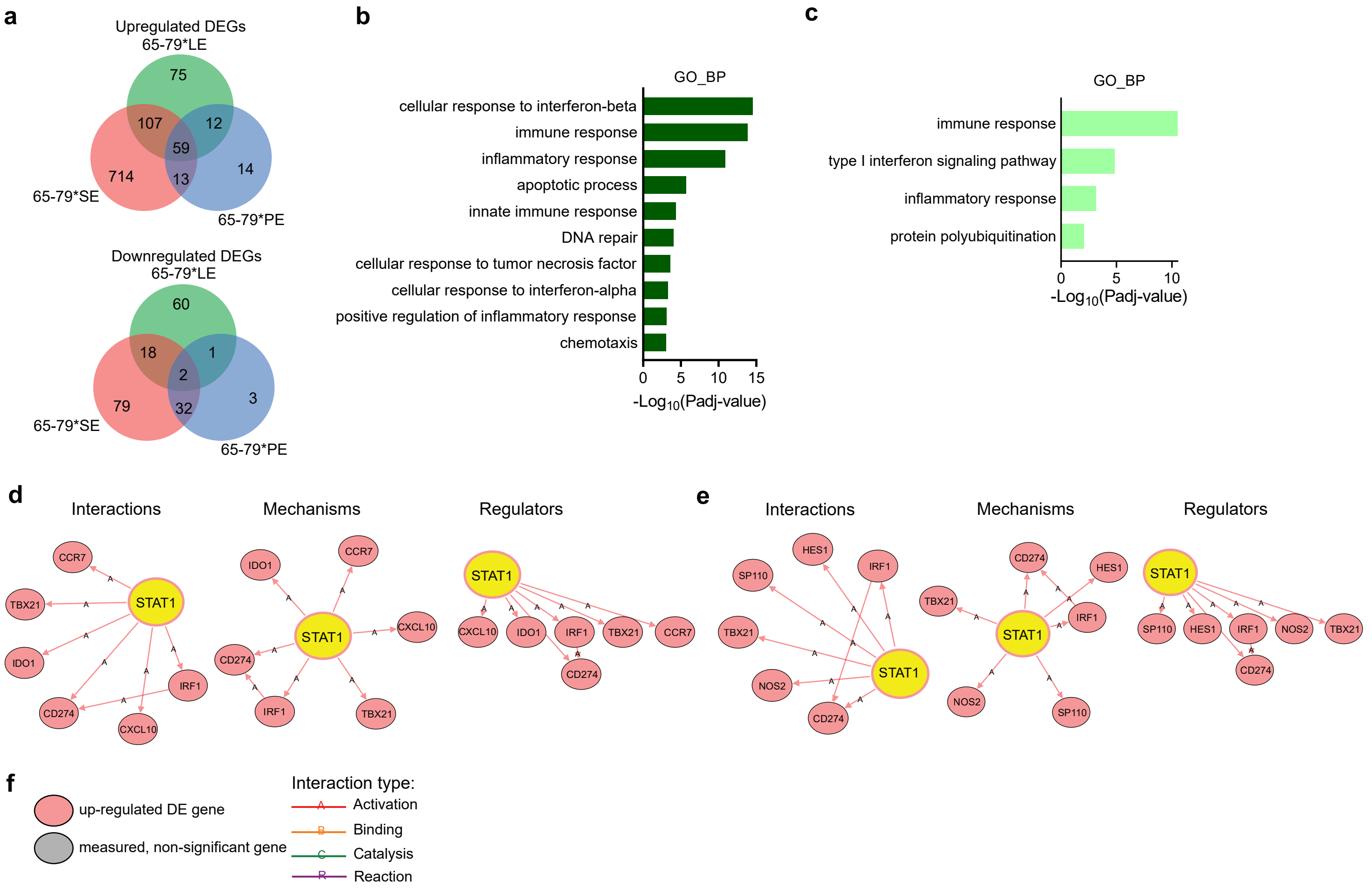

Supplementary Fig. 2: LE-activated transcriptional modulation in THP-1 and RAW 264.7 macrophages (Model B). Related to Fig. 2.

a, Venn diagrams showing DEG comparisons in 65-79*LE-, 65-79*SE- and 65-79*PE-stimulated THP-1 macrophages.
b,**c**, Notable enriched GO-BP terms for 65-79*LE-upregulated DEGs in RAW 264.7 **b**, and THP-1 **c**, macrophages.
d,**e**, Regulatory networks comparing the interactions, mechanisms and regulator modes for STAT1 in THP-1 macrophages activated by 65-79*LE **d**, versus 65-79*SE **e**,.
f, Regulatory network legend (iPG).

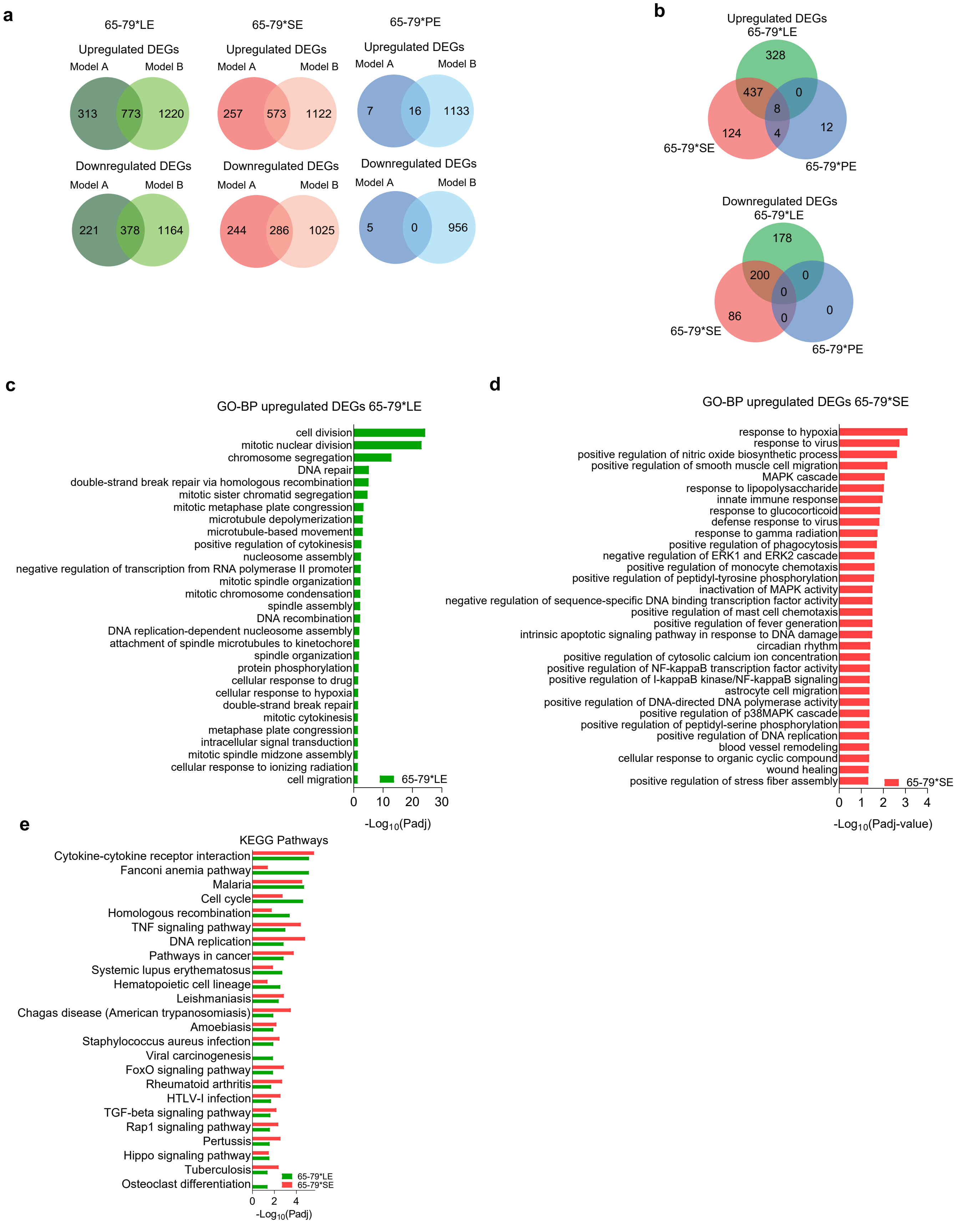

Supplementary Fig. 3: Background information and terminologies. Related to Figs. 1 and 2.

a, Venn diagrams showing comparison of DEGs in Model A and Model B for 65-79*LE, 65-79*SE and 65-79*PE.
b, Venn diagrams showing comparison of DEGs similar between Model A and Model B for 65-79*LE, 65-79*SE and 65-79*PE.
c, d, Unique GO-BP terms enriched for by upregulated DEGs similar between Model A and Model B 65-79*LE and 65-79*SE.
e, KEGG pathway enrichment for upregulated DEGs similar between Model A and Model B for 65-79*LE and 65-79*SE.

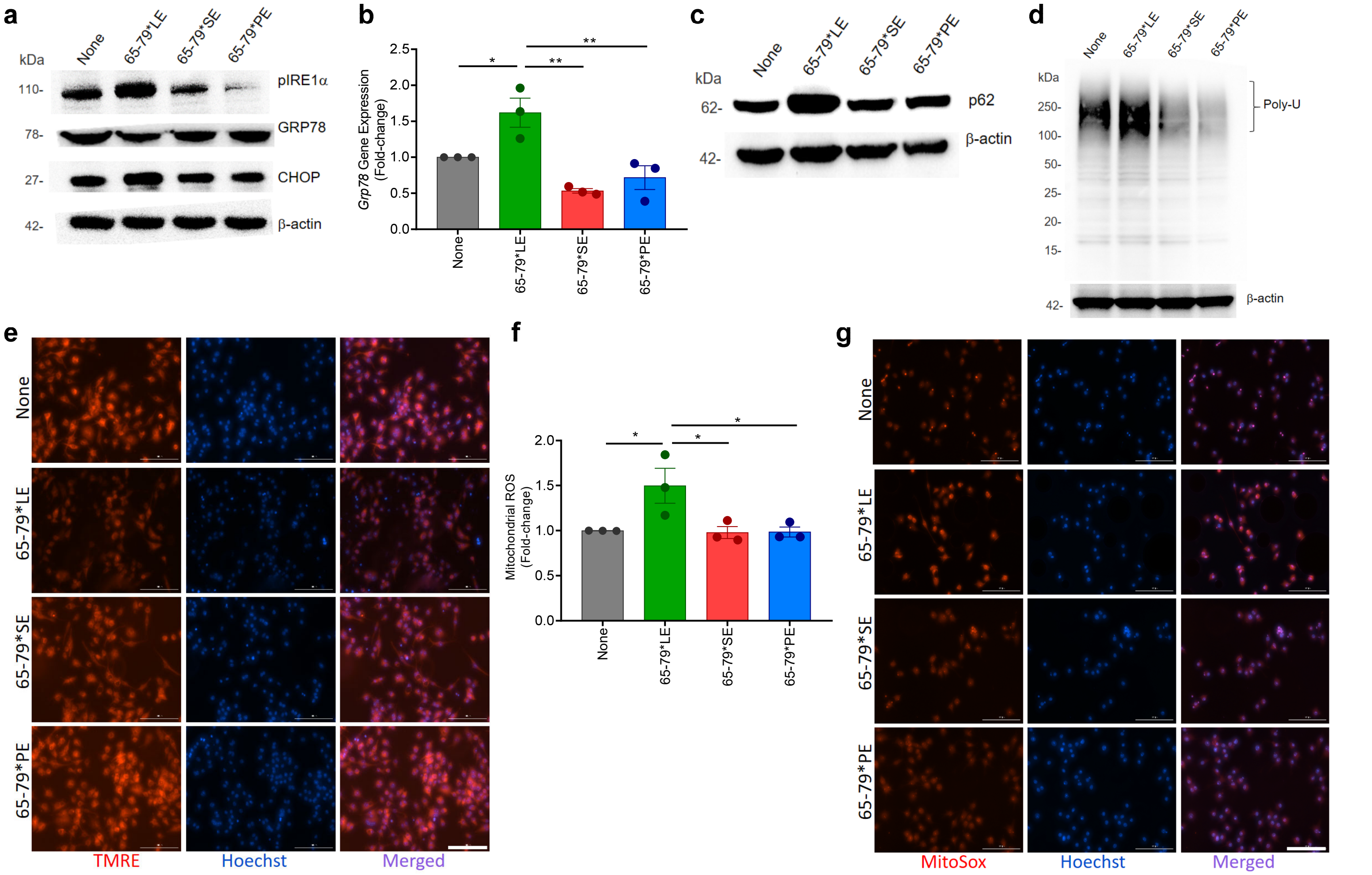

Supplementary Fig. 4: LE-triggered ER stress and mitochondrial dysfunction in mouse RAW 264.7 macrophages. Related to Fig. 3.

a, Representative immunoblots of ER stress markers pIRE1-α, GRP78 and CHOP in RAW 264.7 macrophages exposed to different allelic epitopes in the presence of IFN-γ.
b, qRT-PCR analysis of *Grp78* expression in RAW 264.7 macrophages exposed to different allelic epitopes in the presence of IFN-γ.
c, **d**, Immunoblots of p62 **c**, and poly-ubiquitinated proteins **d**, in RAW 264.7 macrophages stimulated by different allelic epitopes in the presence of IFN-γ.
e, Representative TMRE immunocytochemistry images of allelic epitope-exposed RAW 264.7 macrophages in the presence of IFN-γ. Scale bar=100 μm.
f, Mitochondrial ROS in RAW 264.7 macrophages exposed to allelic epitopes in the presence of IFN-γ and measured by MitoSOX.
g, Representative MitoSOX immunocytochemistry images of allelic epitope-exposed THP-1 macrophages in the presence of IFN-γ. Scale bar=100 μm.
Blots (**a**, **c**, **d**) are representative of 3 independent experiments. Uncropped immunoblots are shown in Supplementary Fig. S9. Bar graphs (**b**, **f**) represent mean ± SEM, one-way ANOVA of repeated measures/Tukey, *$p < 0.05$; **$p < 0.01$; ***$p < 0.001$, ****$p < 0.0001$.

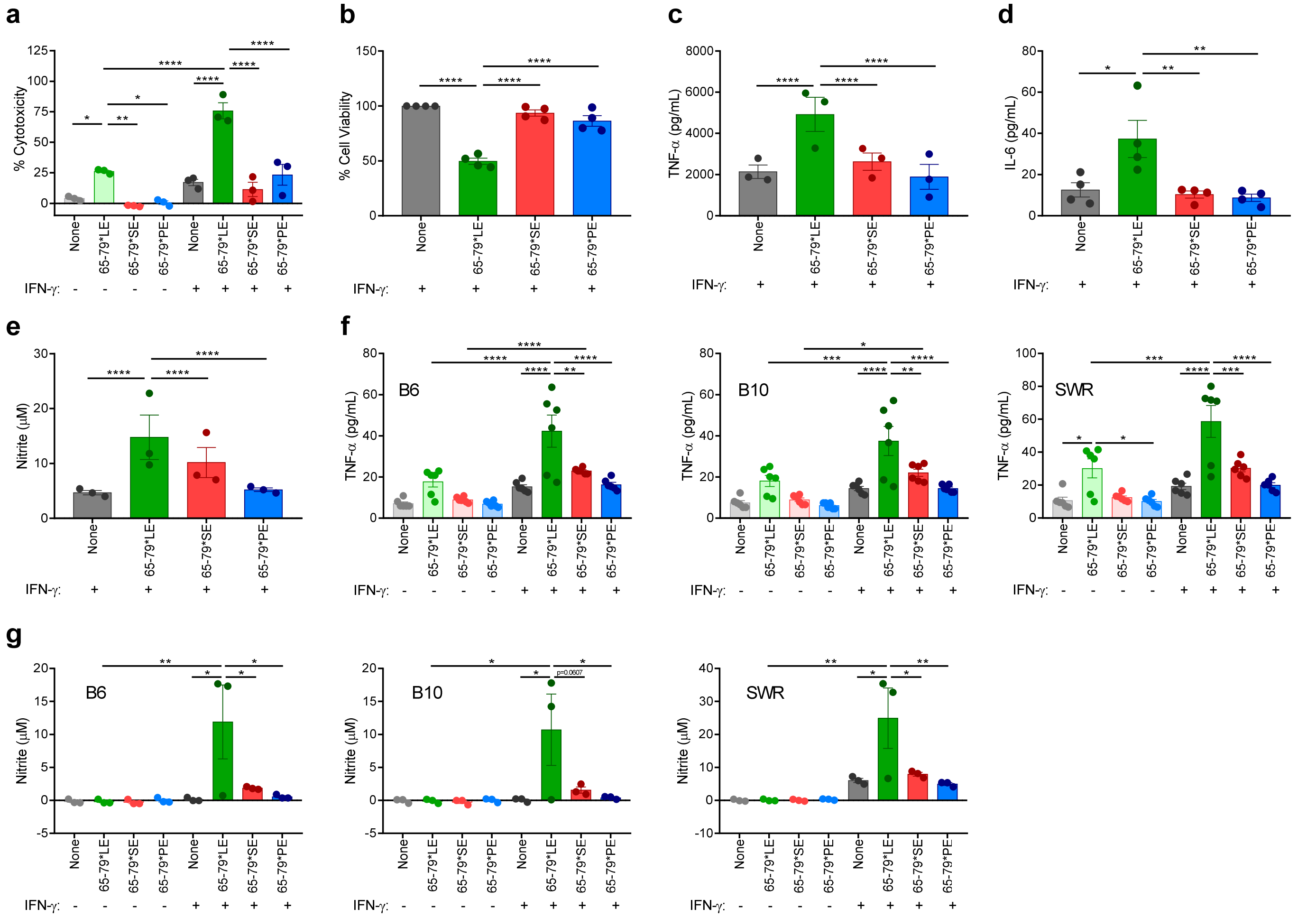

Supplementary Fig. 5: The LE triggers cell death and pro-inflammatory cytokine production in mouse RAW 264.7 macrophages and primary BMDMs from WT mouse strains. Related to Fig. 4.

a, Cell death (n=3) and **b**, viability assessed by MTT (n=4) of RAW 264.7 macrophages treated with different allelic epitopes.
c-e, Levels of pro-inflammatory cytokines TNF-α (n=3) c, and IL-6 (n=4) **d**, as well as nitrite levels (n=3) **e**, in supernatants of allelic epitope-treated RAW 264.7 macrophage.
f, TNF-α and **g**, nitrite supernatant levels in BMDMs derived from control WT mouse strains B6J, B10J and SWR, cultured ex vivo in the presence or absence of IFN-γ (5 ng/mL).
Data represent mean ± SEM. One-way (**b-e**), or two-way (**a**, **f**, **g**) ANOVA of repeated measures/Tukey. *p < 0.05; **p < 0.01; ***p < 0.001, ****p < 0.0001.

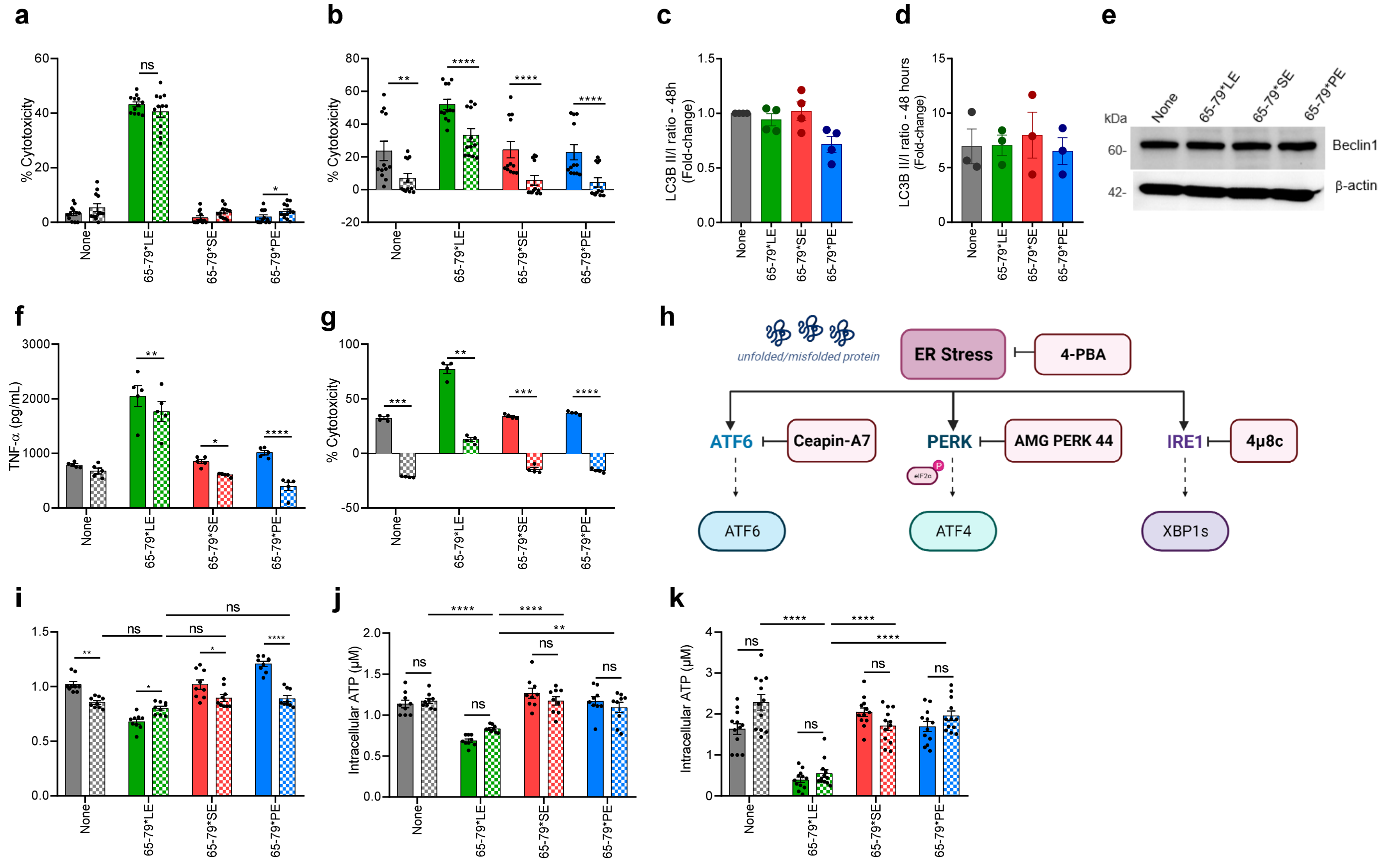

Supplementary Fig. 6: Additional characterization of LE-activated cell death aberrations. Related to Fig. 5 and Fig. 6.

a, The pan-caspase inhibitor ZVAD-FMK (10 μM) does not hinder 65-79*LE-activated cell death in RAW 264.7 macrophages (n=3).
b, Rapamycin (50 nM) has an allele-nonspecific inhibitory effect on 65-79*LE-activated cell death in RAW 264.7 macrophages (n=3).
c, **d**, LC3 II/ LC3 I ratio in allelic epitope-treated RAW 264.7 **c**, (n=4) and THP-1 **d**, (n=3) macrophages.
e, Immunoblot of Beclin1 in different epitope-treated RAW 264.7 macrophages. A representative blot, one of 3 independent experiments.
f, Necrostatin-1 (50 μM) shows a modest, allele-nonspecific inhibitory effect on LE-activated levels of TNF-α in RAW 264.7 macrophage supernatants (n=5).
g, A TNF-α inhibitor [6,7-Dimethyl-3-((methyl-(2-(methyl-(1-(3-trifluoromethyl-phenyl)-1H-indol-3-ylmethyl)-amino)-ethyl)-amino)-methyl)-chromen-4-one] (10 μM) blocks 65-79*LE-activated cell death in an allele-nonspecific fashion.
h, ER stress pathways and their respective inhibitors.
i, ATF6α pathway signaling blocker, Ceapin-A7 (10 μM) shows allele-specific effect on intracellular levels of ATP in RAW 264.7 macrophage supernatants (n=3).
j, **k**, PERK (EIF2AK3) pathway inhibitor, AMG PERK 44 (25 μM) and ER transmembrane protein IRE1 inhibitor, 4μ8C (10 μM) do not rescue intracellular ATP levels in RAW 264.7 macrophages (n=3).
In (**a**, **b**, **f-k**), solid-color and dotted-color bars represent, respectively, absence or presence of inhibitors. Uncropped immunoblots are shown in Supplementary Fig. S9.
Data represent mean ± SEM. Two-way ANOVA of repeated measures/Tukey. *$p < 0.05$; **$p < 0.01$; ***$p < 0.001$, ****$p < 0.0001$.

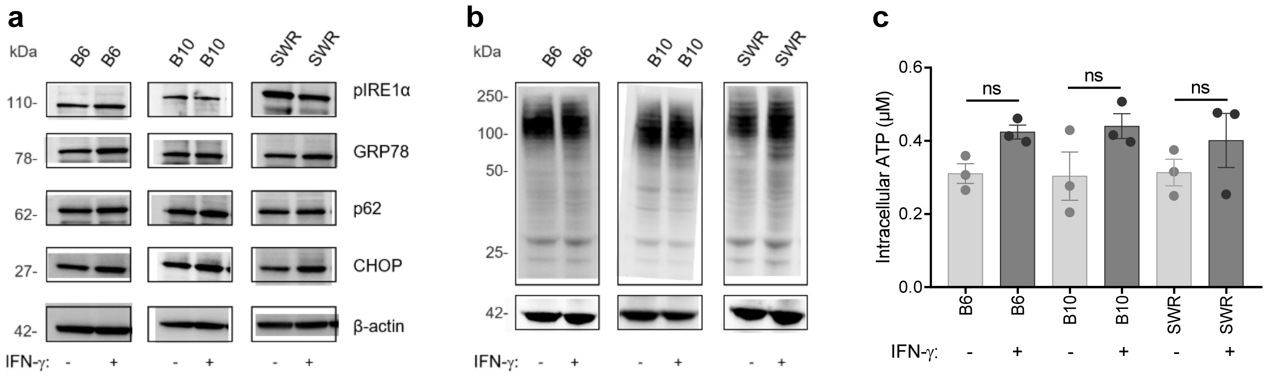

Supplementary Fig. 7: LE-effects in control mice BMDMs Related to Fig. 7.

a, **b**, Immunoblots of ER stress (pIRE1-α, CHOP, GRP78) and proteasomal degradation (p62) markers **a**, and poly-ubiquitinated proteins **b**, in BMDMs derived from WT control mouse strains B6J, B10J and SWR, cultured ex vivo in the presence or absence of IFN-γ (5 ng/mL).
c, Intracellular ATP in BMDMs derived from control mice B6J, B10J and SWR, cultured ex vivo in the presence or absence of IFN-γ (5 ng/mL).
Uncropped immunoblots are shown in Supplementary Fig. S9.
Data represent mean ± SEM. One-way ANOVA (**c**).

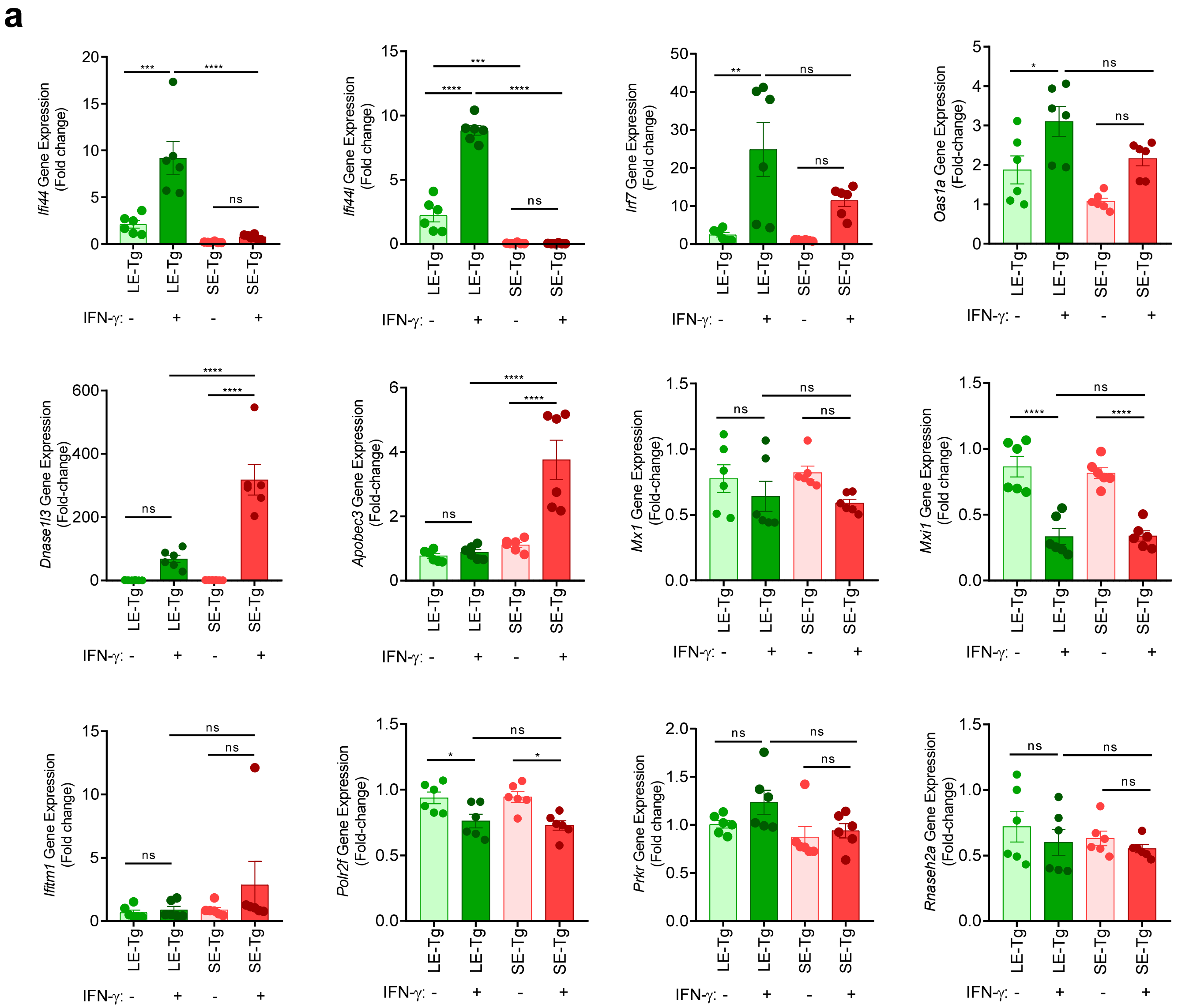

Supplementary Fig. 8: IFN-I gene expression levels in BMDMs from Tg mice. Related to Fig. 7

a, qRT-PCR analysis of the expression levels of salient IFN-I genes in BMDMs derived from transgenic mice (n=6) expressing physiologically folded HLA-DRβ molecules coded by *DRB1*03:01* (LE-Tg) or *DRB1*04:01* (SE-Tg) and cultured *ex vivo* for 24h in the presence or absence of IFN-γ (5 ng/mL). Results represent gene expression relative to LE-Tg without IFN-γ treatment. Data represent mean ± SEM, one-way ANOVA of repeated measures/Tukey, *p < 0.05; **p < 0.01; ***p < 0.001, ****p < 0.0001.

Supplementary Fig. 9: Uncropped blots

Related to Figs. 3,5,6 & 7 and Supplementary Figs. 4S, 6S & 7S.

Figure 3B:

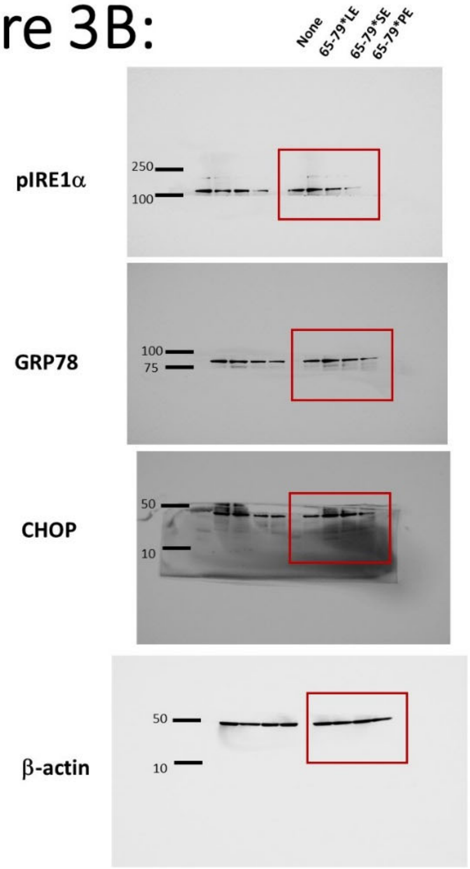

None
65-79*LE
65-79*SE
65-79*PE
pIRE1α
250
100
GRP78
100
75
CHOP
50
10
β-actin
50
10

Figure 3C:

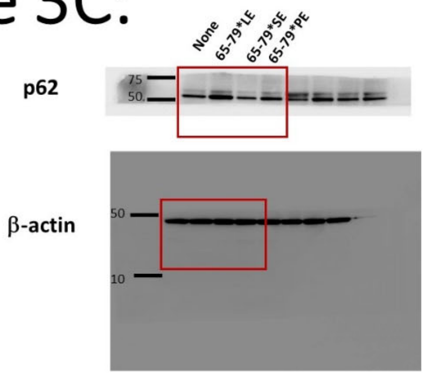

Figure 3D:

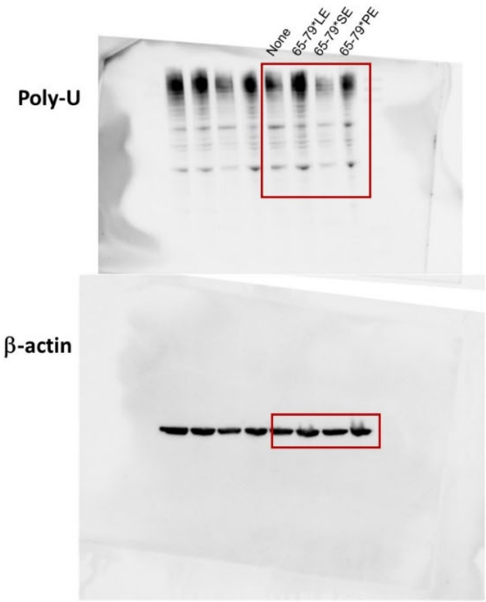

Figure 5D:

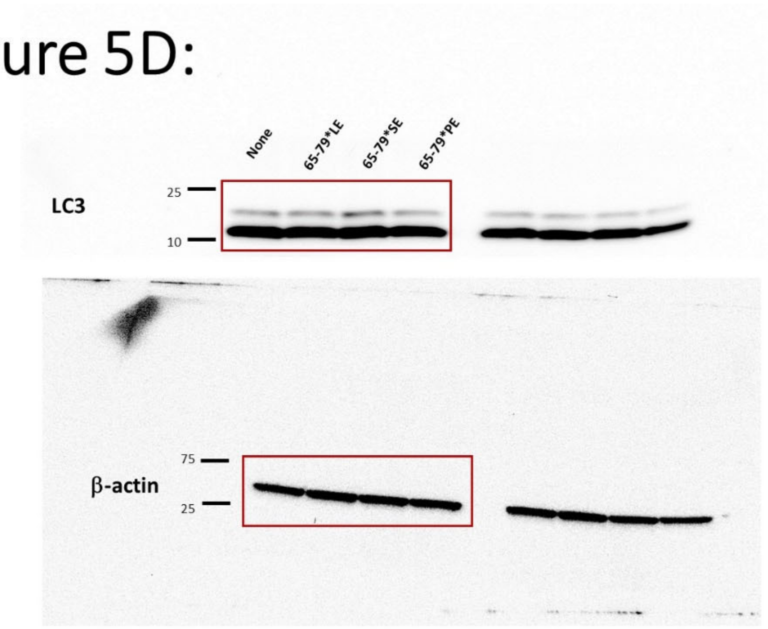

Figure 5E:

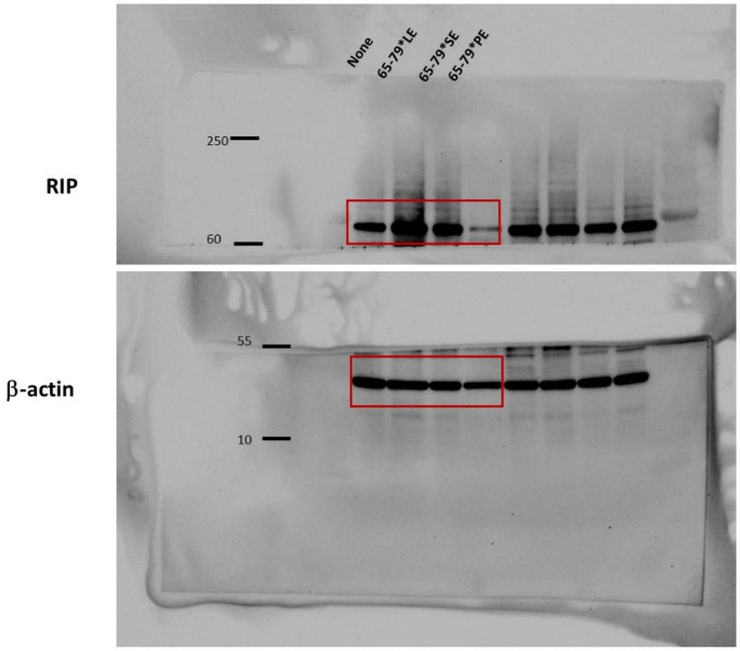

Figure 5f:

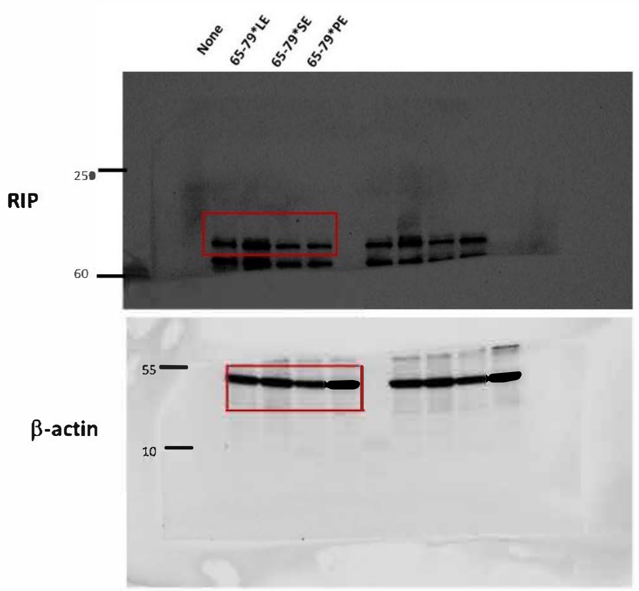

Figure 5g:

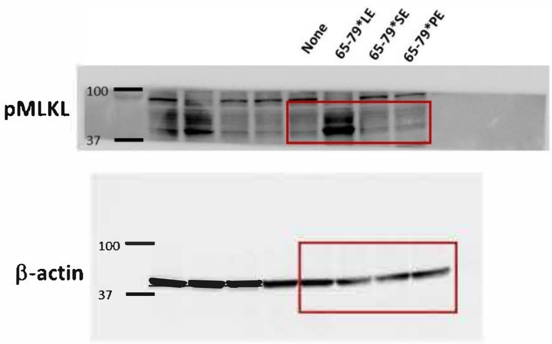

Figure 5h:

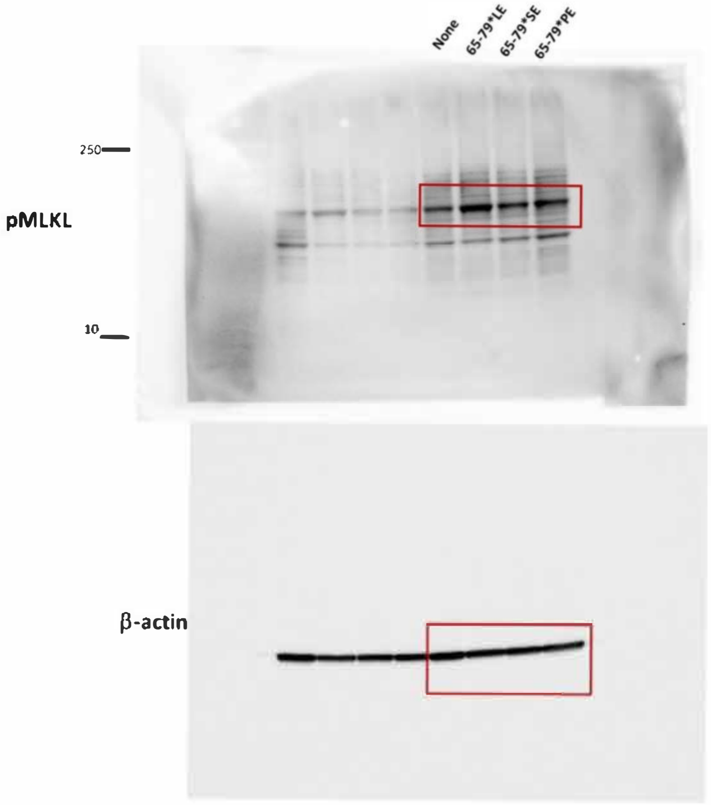

Figure 6a

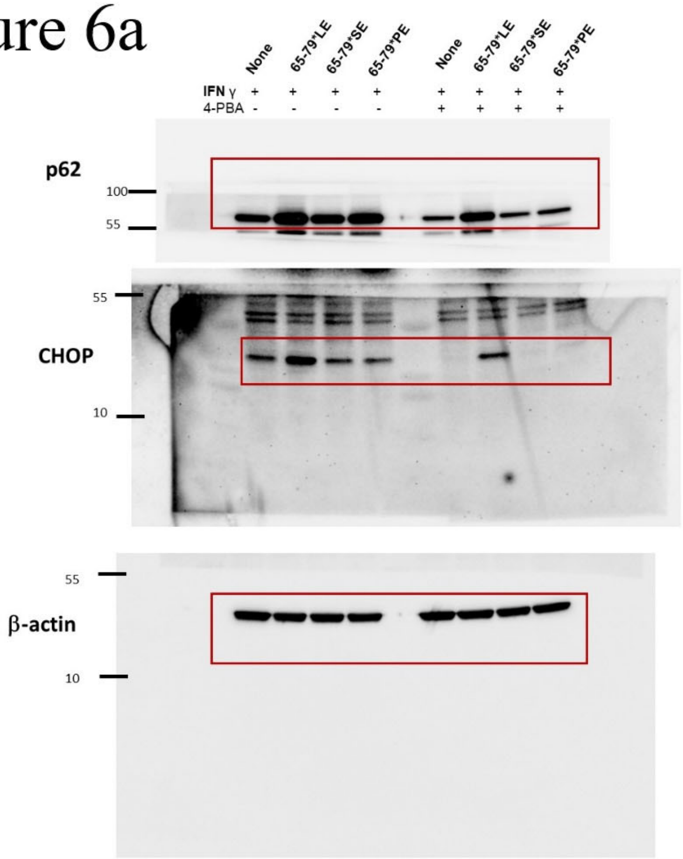

None
65-79*LE
65-79*SE
65-79*PE
None
65-79*LE
65-79*SE
65-79*PE
IFN γ + + + + + + + +
4-PBA - - - - + + + +
p62
100
55
CHOP
55
10
β-actin
55
10

Figure 6E:

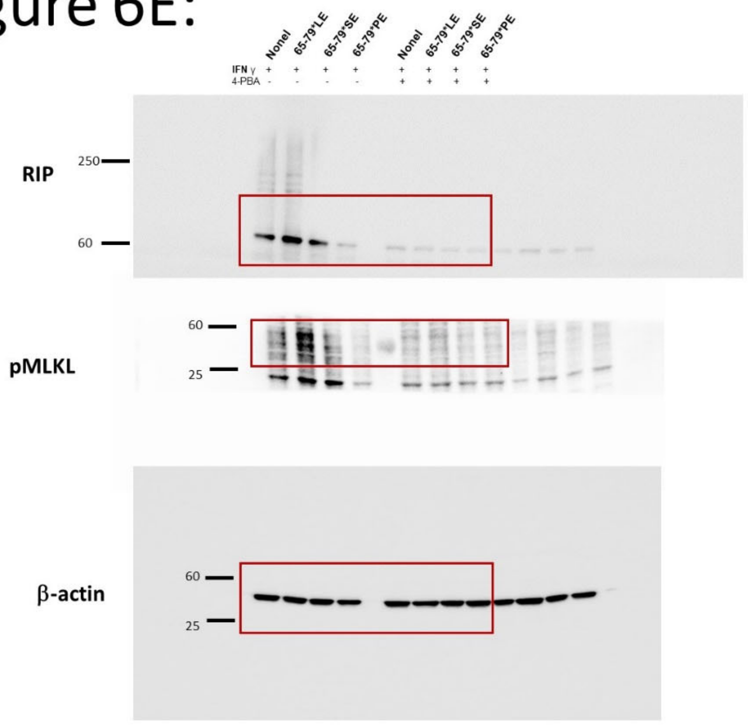

Nonel
65-79*LE
65-79*SE
65-79*PE
Nonel
65-79*LE
65-79*SE
65-79*PE
IFN γ
4-PBA
RIP
250
60
pMLKL
60
25
β-actin
60
25

Figure 7 A:

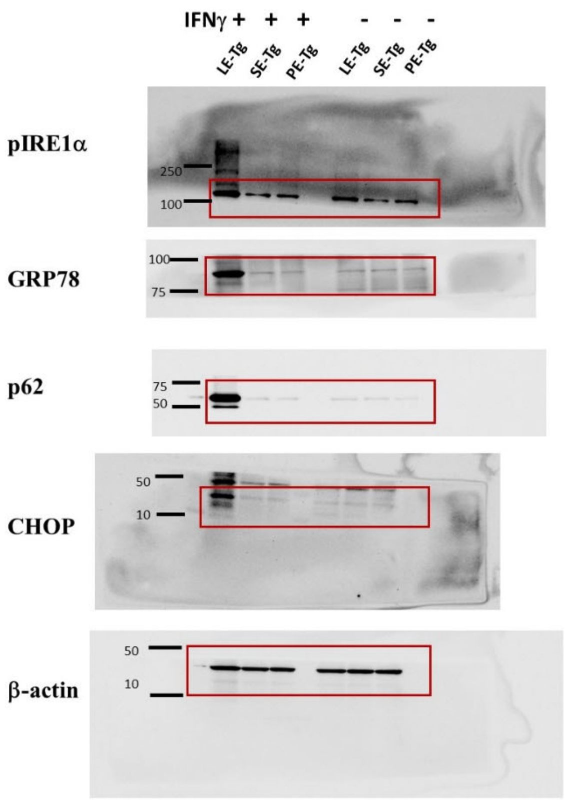

IFNγ + + + - - -
LE-Tg SE-Tg PE-Tg LE-Tg SE-Tg PE-Tg
pIRE1α
250
100
GRP78
100
75
p62
75
50
CHOP
50
10
β-actin
50
10

Figure 7 B:

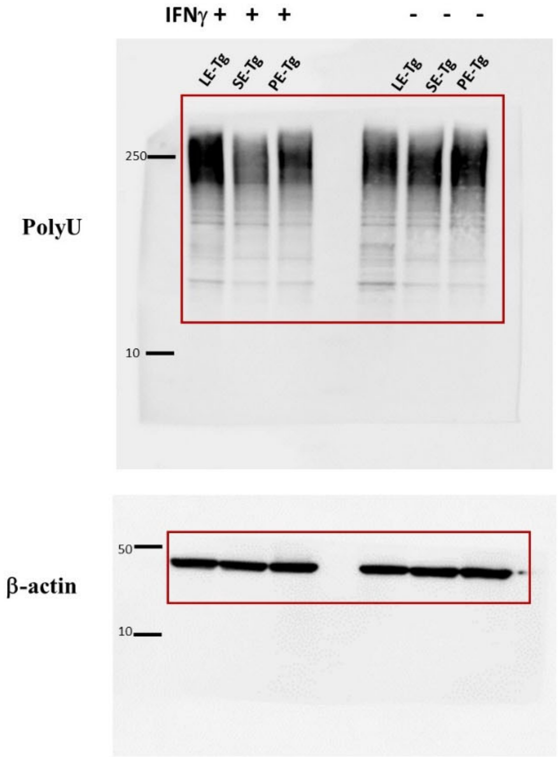

Figure 4S a

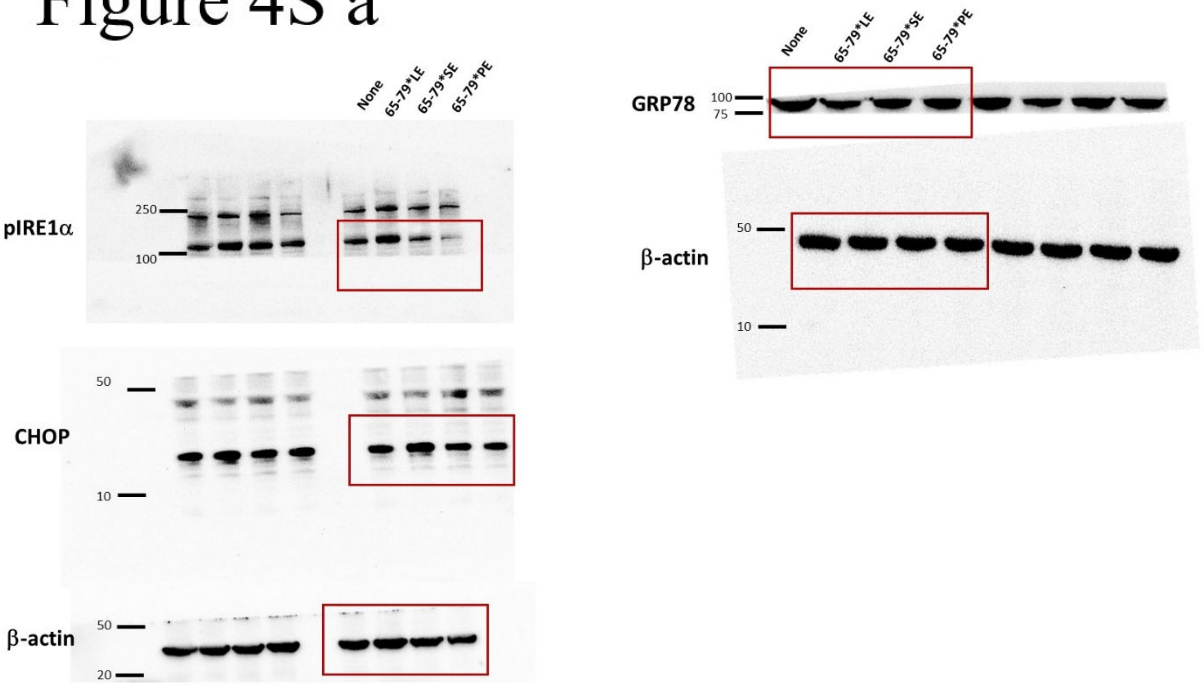
None
65-79*LE
65-79*SE
65-79*PE
pIRE1α
250
100
CHOP
50
10
β-actin
50
20
GRP78
100
75
β-actin
50
10

Figure 4S c

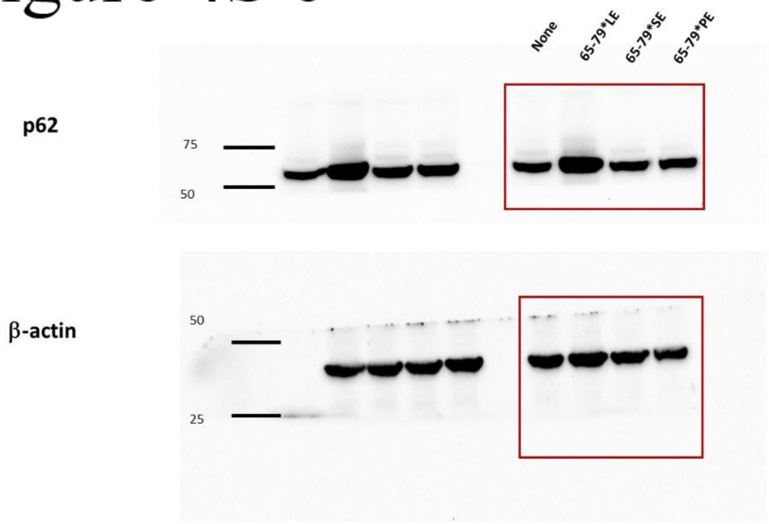

Figure 4S d

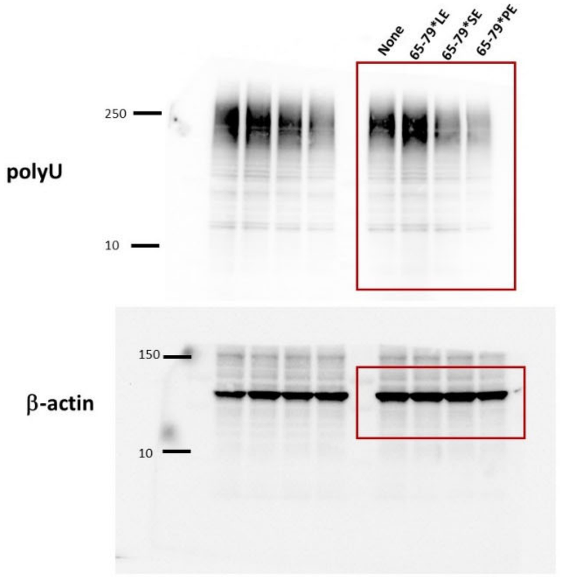

Figure 6S e

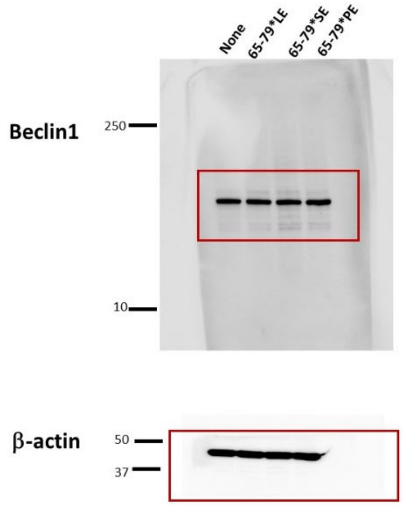

Figure S7 a

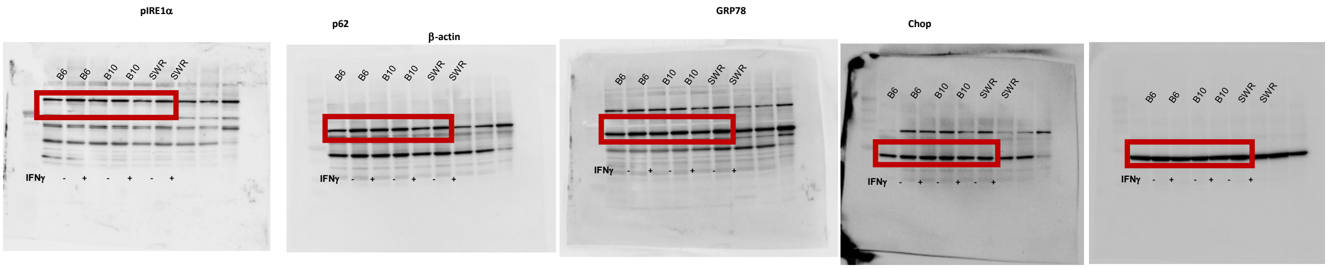

Figure S7 b

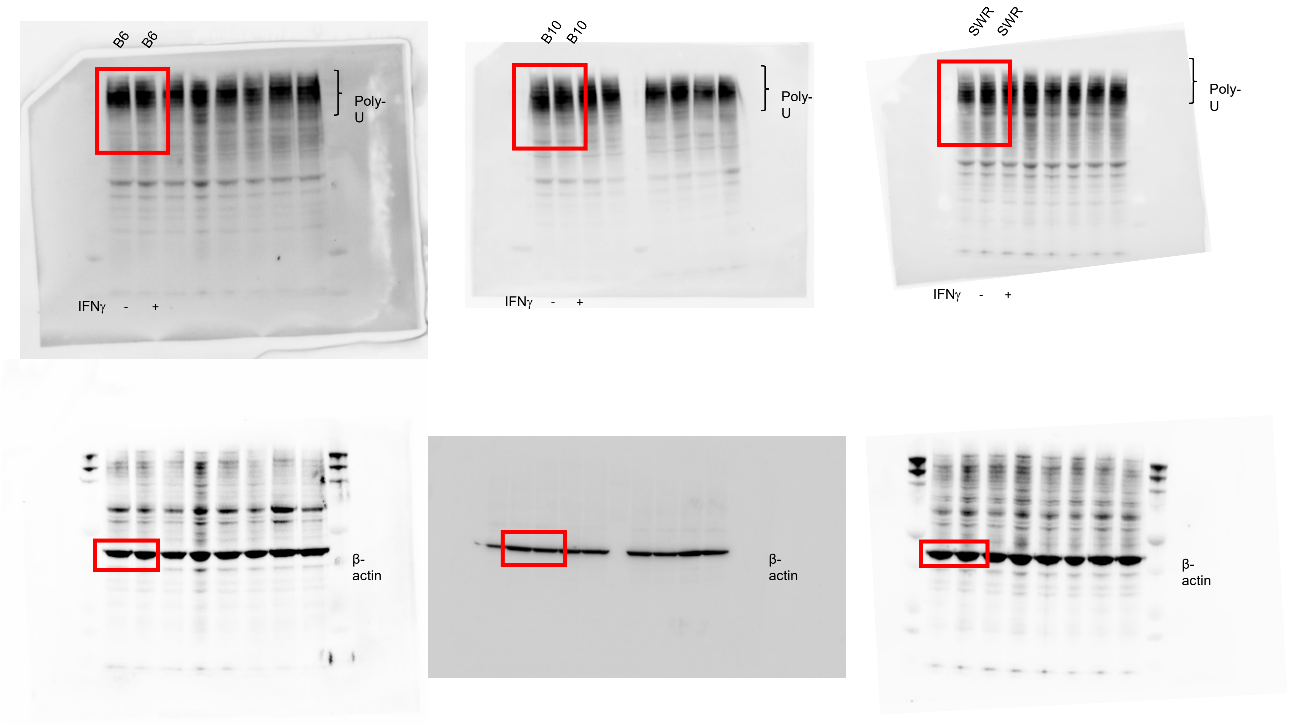

Supplementary Table 1: DEGs with disease relevance in Model A, related to Figure 1

Supplementary Table 1A: Notable SLE-relevant genes modulated by 65-79*LE

Upregulated	FC	Padj	Function
Ccl5	3.97	2.07×10^{-66}	Upregulated in lupus mice and LN urine
Cxcl2	4.62	3.19×10^{-63}	Upregulated in LN; biomarker for active SLE
Pim1	3.35	9.42×10^{-55}	Therapeutic target in LN; STAT-induced signaling
Lig1	2.68	4.08×10^{-49}	DNA damage repair
Myc	4.68	3.32×10^{-46}	Upregulated in SLE; regulates apoptosis
Ccl3	3.32	1.98×10^{-42}	Autoantibody target is SLE; increased in discoid lupus
Dusp1	3.82	2.79×10^{-39}	Regulate T cell-mediated autoimmune responses
Slamf7	2.05	3.29×10^{-34}	Altered expression in SLE
Tnfrsf9	4.07	1.84×10^{-33}	CD137, expressed on activated T cells; associated with enhanced IFN-γ production
Zfp36	2.03	5.95×10^{-33}	Regulates proinflammatory and immune responses; regulates necroptosis
Brca2	2.35	1.39×10^{-30}	DNA damage repair
Chek1	2.82	2.16×10^{-30}	DNA damage response
Timeless	2.24	8.35×10^{-30}	DNA damage repair
Ccl9	2.52	1.25×10^{-29}	Involved in autoimmunity; associated with inflammation
Tnf	2.37	5.68×10^{-29}	Aberrant expression in SLE and autoimmune diseases
Cxcl16	2.71	9.43×10^{-29}	Increased in SLE
Cxcl10	3.66	4.78×10^{-28}	Biomarker of disease severity in LN
Traf1	2.25	4.10×10^{-27}	Polymorphism in SLE
Irf4	3.44	2.29×10^{-25}	Differential expressed in SLE; regulates IFN-driven disease
Dusp4	2.57	6.58×10^{-25}	Overexpressed in T cells of human SLE patients
Smurf1	1.62	6.35×10^{-25}	E3 ubiquitin-protein ligase; plays a role in autoimmunity
Tnfaip3	2.61	9.86×10^{-25}	Deubiquitinating enzyme; susceptibility SNPs in SLE

Downregulated	FC	Padj	Function
Pccb	1.76	3.91×10^{-56}	Plays a role in normal protein processing
Rpn1	1.8	1.27×10^{-54}	Ubiquitin receptor
Scd2	2.77	2.79×10^{-39}	ER enzyme, necessary to prevent ER stress
Erp29	1.51	2.53×10^{-30}	Plays a role in ER stress and the UPR
Hagh	1.56	3.29×10^{-30}	Hydroxyacylglutathione hydrolase, mitochondrial
Pon2	1.74	1.89×10^{-29}	Pro-M2; anti-inflammatory; anti-oxidant; anti-atherogenic
Nucb1	1.9	5.46×10^{-26}	ER protein associated with COX signaling
Marveld1	2.15	5.90×10^{-25}	Reduced Marveld1 increases ROS
Vsir	1.69	9.08×10^{-25}	Reduced expression leads to spontaneous CLE and SLE in mouse
Bcat2	1.98	3.30×10^{-23}	Mitochondrial enzyme
G6pc3	1.51	6.04×10^{-23}	Located in the ER; associated with neutrophil dysfunction/neutropenia
Pdia3	1.95	1.48×10^{-22}	ER protein
Uggt1	1.53	2.69×10^{-22}	Gatekeeper in the ER quality control system
Calr	1.89	4.93×10^{-22}	Defective calreticulin mediated clearance of apoptotic cells in SLE
Arl11	1.52	1.28×10^{-20}	Pro-apoptotic properties
Atg9b	2.34	2.16×10^{-20}	Deficiency potentiates ER stress-associated apoptosis
Aldh18a1	1.5	2.16×10^{-19}	Mitochondrial enzyme
Pnkp	1.5	3.09×10^{-19}	Involved in DNA strand repair

Cyp27a1	1.64	3.55×10^{-19}	Mitochondrial enzyme
Jmjd8	1.74	4.38×10^{-19}	ER protein
Msmo1	1.61	4.44×10^{-18}	Localized in the ER
Bid	1.64	6.28×10^{-18}	Sensor of cellular stress and DNA damage
Jagn1	1.69	6.86×10^{-18}	Induced in ER stress response
Hsp90b1	2.15	7.26×10^{-18}	GRP94, ER chaperone
Smim14	1.89	8.65×10^{-18}	Possibly plays a role in ER Ca^{2+} homeostasis and ER stress
Prkcsh	1.74	8.06×10^{-17}	Subunit of glucosidase II which is ER located

Supplementary Table 1B: Notable RA-relevant genes modulated by 65-79*SE

Upregulated	FC	Padj	Function
Pim1	4.00	1.64×10^{-72}	Regulates pro-inflammatory cytokines in RA
Ccrl2	4.46	3.95×10^{-59}	Upregulated in RA
Pgf	13.14	9.69×10^{-59}	Pro-RA; pro-angiogenesis
Ptpn14	7.51	8.36×10^{-56}	Overexpression in RA FLS
Igf1	2.57	4.11×10^{-49}	Bone modelling
Traf1	2.88	1.96×10^{-46}	RA risk locus
Gpr137b	1.83	8.20×10^{-45}	RA associated locus
Tnfrsf9	4.99	3.32×10^{-44}	CD139, potential RA treatment target
Itgb2	1.90	1.99×10^{-41}	CD18, required for development of inflammatory arthritis
Zfp36	2.20	3.22×10^{-41}	Increased expression in RA; polymorphisms associated with RA; treatment target in RA
Upp1	4.35	5.71×10^{-38}	Increased expression in CIA rat model
Cxcl2	3.20	6.64×10^{-37}	Increased in RA
Cxcl16	3.07	2.08×10^{-36}	Increased expression in RA
Ccl9	2.69	5.26×10^{-34}	Can activate OCs
Tnfaip3	2.98	7.31×10^{-32}	RA risk locus
Ccl22	5.93	2.67×10^{-31}	Potential therapeutic target for RA; expressed in RA synovium
Cd52	2.13	4.26×10^{-30}	Treatment target in RA
Cd83	3.10	5.37×10^{-29}	RA-associated
Dusp4	2.71	1.03×10^{-27}	Pro-angiogenic; pro-Th17 polarization
Pdpn	2.20	2.26×10^{-26}	Implicated in RA; expressed in RA synovium
Adamts1	2.15	7.38×10^{-26}	Involved in angiogenesis
Ninj1	1.51	8.26×10^{-26}	Positively regulates OC development
Rgs1	2.91	1.37×10^{-25}	Involved in inflammation and angiogenesis in RA rats
Rgcc	4.44	1.59×10^{-25}	Dysregulated in RA joint microenvironment
Relb	1.81	7.56×10^{-25}	NF-κB factor associated with RA
Tnip1	1.63	1.62×10^{-24}	Increased expression in RA

Downregulated	FC	Padj	Function
Dock2	1.59	1.24×10^{-65}	Immune regulatory
Scd2	3.52	7.51×10^{-60}	ER enzyme, necessary to prevent ER stress
Sec63	1.53	1.75×10^{-36}	Part of ER protein translocation apparatus
Ero1lb	1.68	1.02×10^{-32}	ER associated markers
Cyb5b	1.64	9.38×10^{-26}	CYB5 mitochondrial isoform
Pon2	1.66	9.03×10^{-25}	Pro-M2; anti-inflammatory; anti-oxidant

Bet1	1.59	4.52×10^{-20}	Involved in maintenance of mitochondrial functions
Scd1	2.51	6.50×10^{-20}	ER enzyme prevents ER stress
Tmem97	1.53	5.12×10^{-19}	Located in ER membrane; activates mitochondrial superoxide pathway
Irgm2	1.56	6.12×10^{-18}	Inhibits caspase 11
Vcp	1.56	9.98×10^{-18}	Facilitates polypeptide degradation
Eif2ak3	1.62	1.20×10^{-17}	PERK, part of the UPR
Cd33	1.74	5.17×10^{-16}	Siglec-3, immune-modulatory receptor
Osbp	1.5	7.20×10^{-16}	Regulates ER-Golgi membrane contact formation
Vmac	2.24	1.14×10^{-15}	E3 ubiquitin ligase; involved in STING signaling
Cyp51	1.64	1.26×10^{-15}	Anti-inflammatory
Txndc5	1.8	1.03×10^{-14}	Anti-oxidative stress
Rnf145	1.63	1.06×10^{-14}	Ubiquitin ligase

Supplementary Table 2: DEGs with disease relevance in Model B, related to Figure 2

Supplementary Table 2A: Notable SLE-relevant genes modulated by 65-79*LE

Upregulated	FC	Padj	Function
Cd274	9.05	1.32×10^{-189}	Increased in SLE patients
Cd36	10.58	9.36×10^{-165}	Candidate hub gene in LN; expressed on the majority of monocytes in SLE
Rsad2	12.82	2.06×10^{-103}	Increased expression in SLE
Dio2	14.22	4.94×10^{-95}	Associated with necroptosis
Cxcl10	17.98	3.86×10^{-66}	Biomarker of disease severity in LN
Ifit3	7.75	4.12×10^{-41}	Increased in SLE
Nos2	12.87	1.05×10^{-39}	M1 marker; involved in SLE pathogenesis
Cd69	11.51	3.00×10^{-37}	Can act as a proinflammatory receptor
Myc	7.98	1.17×10^{-36}	Upregulated in SLE
Il27	7.72	1.15×10^{-34}	Augmented in SLE; risk locus for SLE
Rasd2	8.9	7.76×10^{-20}	Main hub gene in LN
Gpr18	8.09	7.98×10^{-19}	Expressed in SLE monocytes
Slc7a2	13.77	1.41×10^{-17}	Required for NO production
Ms4a4c	8.25	3.18×10^{-17}	Upregulated in murine SLE models
Ccl8	7.59	6.28×10^{-12}	Increased in SLE patients with active renal disease

Downregulated	FC	Padj	Function
Dhcr24	9.86	1.80×10^{-124}	Anti-ER stress induced apoptosis
Acaa2	3.67	2.80×10^{-105}	A mitochondrial enzyme; decreased in SLE
Atg9b	4.35	7.42×10^{-67}	Anti-ER stress-associated apoptosis
Alox5	4.95	3.76×10^{-57}	Anti-mitochondrial apoptotic pathway
Nlrp10	3.94	1.00×10^{-55}	Protects against kidney damage; inhibits IL-1β secretion; inhibits NF-κB activation and inhibits apoptosis
Fkbp11	4.22	3.78×10^{-31}	ER stress/UPR gene; anti-inflammation induced apoptosis
Lrrc1	4.3	7.19×10^{-21}	LRRC1-/- cells deregulate Wnt/β-catenin signaling
Cd24a	3.24	2.40×10^{-14}	Triggers caspase-dependent apoptosis; high levels inversely correlated with SLEDAI score
Fhit	3.77	1.32×10^{-11}	Pro-apoptosis
Rab15	4.22	1.61×10^{-11}	Involved in endocytic receptor recycling
Asb10	4.22	3.68×10^{-10}	Functions in ubiquitin-mediated degradation pathways
Map2k6	3.41	1.07×10^{-7}	Pro-survival kinase

Supplementary Table 2B: Notable autoimmune disease-relevant genes modulated by 65-79*PE

Upregulated	FC	Padj	Function
Csf1	6.54	1.91×10^{-32}	MCSF; M2 macrophage associated
Bglap2	7.19	4.93×10^{-30}	Anti-bone resorption
Trim30d	6.87	6.63×10^{-12}	Inhibits NF-κB

Downregulated	FC	Padj	Function
Ndrg4	5.14	1.25×10^{-100}	Induced by TNFα through NF-κB

Ctsk	6.57	2.22×10^{-63}	Osteoclast marker
Mustn1	6.1	6.77×10^{-47}	Over-expressed in arthritis and muscle diseases
Deptor	5.65	2.63×10^{-40}	Involved in mTOR signaling
S100a4	4.27	1.12×10^{-39}	Pro-inflammatory; over-expressed in SLE; biomarker for LN
St6gal1	4.03	1.42×10^{-38}	An SLE disease severity marker
Ndrg2	2.93	5.10×10^{-29}	Pro-inflammatory
Gpr183	4.83	1.14×10^{-27}	Regulates innate and adaptive immunity
Jun	3.03	4.75×10^{-26}	Over-expressed in synovial cells; pro-arthritic in mice
Timp2	4.39	4.74×10^{-25}	Increased in SLE
Cx3cr1	8.83	1.02×10^{-23}	Over-expressed in LN
Tnfrsf9	4.17	8.61×10^{-20}	Autoimmunity-associated locus; over-abundant in RA; therapeutic target in arthritic mice; RA severity locus in African Americans; pro-osteoclastic
Trem2	2.93	1.62×10^{-19}	Regulates OC formation; increased in RA
Rasgrp3	3.56	8.12×10^{-18}	Correlated with SLE disease activity
F10	4.54	8.40×10^{-18}	Factor X, potential key role in RA progression
Slc9b2	7.61	9.46×10^{-16}	Induced by NF-κB
Card14	3.62	6.10×10^{-15}	Induces skin inflammation
Mmp9	2.95	1.01×10^{-14}	Inflammation marker Increased in SLE; SLE-risk locus
Col4a1	4.14	1.39×10^{-14}	Autoantigen in SLE
Clu	4.4	2.96×10^{-13}	Upregulated in inflammatory myopathies
Frat2	4.45	7.94×10^{-12}	Wnt signaling activator
Serpinf1	3.94	6.83×10^{-10}	Mediator of NLRP3 inflammasome
Frat1	3.18	9.02×10^{-10}	Positive regulator of Wnt signaling
F7	3.96	1.05×10^{-8}	Factor VII, plays a role in the pathogenesis of RA; NF-κB and IL-8 activator; pro-angiogenic
Fabp4	3.19	2.97×10^{-8}	Increased levels in RA; pro-angiogenic
Scg2	3.52	1.12×10^{-7}	Pro-angiogenic

Supplementary Table 3: SLE relevant DEGs shared between mouse RAW 264.7 and human THP-1 macrophages, related to Figure 2

	RAW 264.7		THP-1		
	FC	Padj	FC	Padj	Relevant roles/functions
Psmb9	7.89	1.22×10^{-174}	1.98	4.14×10^{-3}	Upregulated in lupus skin
Pim1	5.00	3.40×10^{-117}	3.64	1.50×10^{-2}	Proposed therapeutic target for LN
Irf1	5.00	1.81×10^{-105}	4.16	1.46×10^{-3}	Induces target gene expression in SLE
Rsad2	1.28	2.06×10^{-103}	2.61	3.98×10^{-2}	Upregulated in SLE; IFN signature gene
Stat1	6.42	1.76×10^{-97}	4.31	7.70×10^{-4}	Transduces type I and II IFN signaling; overexpressed in SLE
Prdx5	3.13	7.63×10^{-81}	1.62	2.53×10^{-2}	Differentially methylated in SLE
Dusp1	12.80	2.01×10^{-73}	2.60	4.96×10^{-2}	Regulates T cell-mediated autoimmune responses
Cxcl10	1.80	3.86×10^{-66}	4.00	1.05×10^{-2}	Upregulated and proposed as biomarker for active human SLE
Psme1	4.61	4.35×10^{-60}	1.87	3.00×10^{-3}	Proteasome activator complex subunit; IFNα-inducible gene
Psmb10	2.78	3.78×10^{-55}	1.82	1.29×10^{-2}	Immunoproteasome
Cxcl16	5.59	7.92×10^{-54}	2.50	4.66×10^{-2}	Increased in SLE; biomarker for disease severity in LN
Stat2	3.13	1.80×10^{-41}	2.39	2.49×10^{-2}	Type I IFN-inducible gene; constitutively activated in SLE patients
Cxcl9	1.68	2.69×10^{-41}	2.43	4.67×10^{-11}	Overexpressed in cutaneous lupus in correlation with disease activity
Clec2d	8.26	3.20×10^{-40}	2.03	2.54×10^{-2}	Induces IFN-γ production
Nbn	2.21	8.73×10^{-35}	1.55	5.20×10^{-5}	Potential risk locus for SLE
Dusp5	18.00	6.41×10^{-33}	3.74	1.10×10^{-2}	Regulates T cell-mediated autoimmune responses
Psme2	1.98	3.31×10^{-31}	1.81	4.80×10^{-2}	IFNα-inducible gene
Otud1	3.73	1.95×10^{-25}	1.68	4.76×10^{-2}	Deubiquinase; mutated in SLE
Nampt	1.64	3.16×10^{-24}	2.79	2.88×10^{-2}	Increased in SLE patients
Il15	3.09	3.91×10^{-21}	3.05	2.55×10^{-2}	Upregulated in SLE and LN patients
Parp9	1.52	2.96×10^{-20}	2.23	1.07×10^{-2}	DNA damage repair; upregulated in SLE; differentially methylated in SLE
Ccl22	4.80	2.01×10^{-19}	2.36	4.76×10^{-2}	Risk locus for SLE
Lhfpl2	2.78	1.59×10^{-13}	1.96	1.49×10^{-2}	SLE Meta Signature gene
Parp14	5.59	1.44×10^{-12}	1.93	4.44×10^{-2}	Acts on M1 polarization downstream of STAT1
Dtx3l	1.69	3.99×10^{-8}	1.80	1.89×10^{-2}	Cooperates with Parp9 in DNA repair; E3 ubiquitin ligase
Scarf1	1.70	4.63×10^{-5}	2.91	3.64×10^{-2}	Induces necroptosis (RIPK1/RIPK3-dependent PARP-1 activation)
Tnfsf10	2.19	5.71×10^{-4}	2.82	1.49×10^{-2}	Upregulated in LN
Cxcr3	- 4.19	6.17×10^{-21}	2.91	1.87×10^{-2}	Cxcl9-Cxcl10 receptor; plays a role in murine LN
Svip	- 2.08	9.27×10^{-6}	2.13	3.13×10^{-2}	Inhibitor of endoplasmic reticulum-associated degradation (ERAD)

Supplementary Table 4: Selected URs for Model A and B, related to Figures 1 and 2

Supplementary Table 4A: Top URs activated by 65-79*LE and 65-79*SE in RAW 264.7 macrophages in Model A

UR	Padj (LE)	Padj (SE)	Relevance
Pclaf	3.56×10^{-6}	5.86×10^{-4}	PCNA; elicits autoimmune responses in SLE; autoantibody target specific for SLE
Il1b	4.02×10^{-6}	8.99×10^{-6}	Increased in SLE
Egf	8.94×10^{-6}	1.09×10^{-3}	Egfr signaling is involved in LN
Egfr	1.72×10^{-5}	1.43×10^{-3}	Egfr signaling is involved in LN
Tslp	1.84×10^{-5}	2.99×10^{-5}	Suggested in SLE nephritis
Tnf	2.82×10^{-5}	1.70×10^{-3}	Increased in SLE and LN
Trp53	2.82×10^{-5}	1.70×10^{-3}	Increased and autoantibody target in SLE
Ccl2	4.02×10^{-5}	1.09×10^{-3}	Therapeutic target in LN
Tlr4	4.67×10^{-5}	2.51×10^{-3}	Increased in SLE
Il33	1.15×10^{-4}	4.77×10^{-3}	Risk locus in SLE; increased in SLE
Tnfsf11	2.41×10^{-3}	1.81×10^{-3}	RANKL; increased in SLE
Csf3	2.69×10^{-3}	8.27×10^{-3}	Enhances OC activity; increases bone resorption in vivo; increases SLE disease activity and renal involvement
Uba52	1.71×10^{-4}	1.43×10^{-3}	Ubiquitin-60S ribosomal protein L40; source of ubiquitin
Rps27a	5.40×10^{-4}	1.81×10^{-3}	Ubiquitin-40S ribosomal protein S27a; source of ubiquitin
Ubc	7.36×10^{-4}	1.39×10^{-2}	Poly-ubiquitin C; encodes poly-ubiquitin
Mcm9	7.80×10^{-4}	2.51×10^{-3}	Involved in dsDNA repair
Stn1	1.98×10^{-3}	1.49×10^{-2}	Part of CST complex; contributes to telomere maintenance
F2	2.69×10^{-3}	8.27×10^{-3}	Prothrombin precursor
Meiob	2.69×10^{-3}	8.27×10^{-3}	Binds ssDNA during meiosis
Clock	2.73×10^{-3}	8.27×10^{-3}	Regulates circadian rhythm

Supplementary Table 4B: Top URs activated by 65-79*LE in THP-1 macrophages in Model B

Upstream regulator	Padj (LE)	Padj (SE)	Padj (PE)	Relevance to SLE
IRF9	9.34×10^{-6}			Type 1 IFN regulated transcription factor
IFNG	1.23×10^{-5}	1.42×10^{-2}	1.01×10^{-5}	Mediates SLE pathogenesis; Increased in RA
STAT2	3.43×10^{-5}			Increased in SLE
STAT1	2.99×10^{-3}	2.83×10^{-2}		Increased in SLE; Increased in RA
IFNA1	3.36×10^{-2}		3.93×10^{-2}	Type 1 IFN, increased in SLE

Supplementary Table 5: Materials and reagents

Reagent or Resource	Source	Catalog numbers
**Antibodies (RRID#)**		
Anti-C3, FITC conjugated; (RRID: AB_2891133)	Immunology Consultants Laboratory (Tigard, OR)	Cat# GC3-90F-Z
Anti-Mouse CHOP (1:1000); (RRID:AB_2089254)	Cell Signaling Technologies (Danvers, MA)	Cat# 2895
Anti-Rabbit IRE1α (1:1000); (RRID:AB_10145203)	Novus Biologicals (Littleton, CO)	Cat# NB100-2323SS
Anti-Mouse BiP/Grp78 (1:1000); (RRID:AB_398292)	BD Biosciences (Franklin Lakes, NJ)	Cat# 610979
Anti-Mouse β-actin (1:4000);(RRID:AB_399901)	BD Biosciences (Franklin Lakes, NJ)	Cat# 612657
Anti-mouse Mono- and polyubiquitinylated conjugates monoclonal (FK2) (WB 1:1000); (ICC 1:200) (RRID:AB_10541840)	Enzo Life science (Farmingdale, NY)	Cat# BML-PW8810
Purified Mouse Anti-RIP (1:1000);(RRID:AB_397831)	BD Biosciences (Franklin Lakes, NJ)	Cat# 610458
Anti-Rabbit Recombinant Anti-MLKL (phospho S345) (1:1000); (RRID:AB_2687465)	Abcam (Cambridge, United Kingdom)	Cat# ab196436
Anti-Rabbit SQSTM1/p62 (1:1000);(RRID:AB_10624872)	Cell Signaling Technologies (Danvers, MA)	Cat# 5114S
Anti-Rabbit LC3B (1:1000);(RRID:AB_915950)	Cell Signaling Technologies (Danvers, MA)	Cat# 2775S
Anti-rabbit IgG, HRP linked; (1:5000);(RRID:AB_2099233)	Cell Signaling Technologies (Danvers, MA)	Cat# 7074S
Anti-mouse IgG HRP-linked; (1:8000);(RRID:AB_772210)	GE healthcare Lifesciences (Chicago, IL)	Cat# NA931
Beclin-1 Antibody (1:1000);(RRID:AB_490837)	Cell Signaling Technologies (Danvers, MA)	Cat# 3738
Goat anti-Mouse IgG (H+L) Cross-Adsorbed Secondary Antibody, Alexa Fluor 647; (1:500);(RRID:AB_2535804)	Thermo Fisher (Waltham, MA)	Cat# A-21235
IFNAR2 Monoclonal Antibody (MMHAR-2) 10µg/mL;(RRID:AB_387828)	PBL Assay Science (Piscataway, NJ)	Cat# 21385-1
**Cell Culture**		
Alpha MEM	Gibco (Waltham, MA	Cat# 12561-056
Antibiotics (Pen Strep)	Gibco (Waltham, MA)	Cat# 15140-122
DMEM	Gibco (Waltham, MA)	Cat# 11885-084
DMEM (high glucose)	Gibco (Waltham, MA)	Cat# 11965-092
Fetal Bovine Serum	Corning (Tewksbury, MA)	Cat# 35-015-CV
L-Glutamine (200 mM)	Gibco (Waltham, MA)	Cat# 25030-081
RPMI 1640	Gibco (Waltham, MA)	Cat# 11875-093
Sodium Pyruvate	Gibco (Waltham, MA)	Cat# 11360-070
StemPro™ Accutase™ Cell Dissociation Reagent	Gibco (Waltham, MA)	Cat# A1110501

**Cells**		
L929	ATCC (Manassas, VA)	Cat# CCL-1
RAW 264.7	ATCC (Manassas, VA)	Cat# TIB-71
THP-1 cells	ATCC (Manassas, VA)	Cat# TIB-202
**Peptides**		
65-79*LE	Bioworld (Dublin, OH)	
65-79*SE	Bioworld (Dublin, OH)	
65-79*PE	Genscript (Piscataway, NJ)	
65-79*1501	Bioworld (Dublin, OH)	
65-79*0403	Bioworld (Dublin, OH)	
**Chemicals, Reagents, peptides, and Recombinant Proteins**		
2′,7′-Dichlorofluorescin diacetate (DCFDA)	Sigma (St. Louis, MO)	Cat# D6883
2x Laemmli Sample Buffer	Bio-Rad (Hercules, CA)	Cat# 1610737
4μ8C	Sigma (St. Louis, MO)	Cat# SML0949
AMG PERK 44	Sigma (St. Louis, MO)	Cat# SML3049
Baricitinib	AChemBlock (Hayward, CA)	Cat# G-5743
Bovine Serum Albumin	Sigma (St. Louis, MO)	Cat# A7906
Ceapin-A7	Sigma (St. Louis, MO)	Cat# SML2330
Clarity™ Western ECL Substrate	Bio-Rad (Hercules, CA)	Cat# 170-5060
cOmplete mini EDTA-free	Roche (Indianapolis, IN)	Cat# 11836170001
Hoechst 33342	Invitrogen (Waltham, MA)	Cat# H3570
Methylthiazolyldiphenyl-tetrazolium bromide (MTT)	Sigma (St. Louis, MO)	Cat# M2128
MitoSOX Red	Invitrogen (Waltham, MA)	Cat# M36008
Necrostatin-1 (Nec-1)	Enzo Life science (Farmingdale, NY)	Cat# BML-AP309-0020
Necrosulfonamide (NSA)	TOCRIS (Bristol, UK)	Cat#5025
Novex™ 4-20% Tris-Glycine gels	Invitrogen (Waltham, MA)	Cat# XP04200BOX
Novex™ WedgeWell 16% Tris-Glycine gels	Invitrogen (Waltham, MA)	Cat# XP0016BOX
NuPAGE™ LDS Sample Buffer (4x)	Invitrogen (Waltham, MA)	Cat# NP0007
NuPAGE™ Sample Reducing agent (10x)	Invitrogen (Waltham, MA)	Cat# NP0009
NuPAGE™ 10% Bis-Tris gel	Invitrogen (Waltham, MA)	Cat# NP0301BOX
PBS (Phosphate buffered saline)	Gibco (Waltham, MA)	Cat# 10010-023
Phorbol 12-myristate 13-acetate (PMA)	Sigma (St. Louis, MO)	Cat# P8139
phosSTOP	Roche (Indianapolis, IN)	Cat# 04906845001
Pluronic™ F-127	Invitrogen (Waltham, MA)	Cat# P3000MP
ProLong™ Diamond Antifade Mountant with DAPI	Invitrogen (Waltham, MA)	Cat# P36966
PVDF Western Blotting Membrane	Roche (Indianapolis, IN)	Cat# 03010040001
Rapamycin	Enzo Life science (Farmingdale, NY)	Cat# BML-A275-0005
Recombinant Human IFN-γ	Peprotech (Rocky Hill, NJ)	Cat# 300-02
Recombinant Murine IFN-γ	Peprotech (Rocky Hill, NJ)	Cat# 315-05
Rhod-2, AM, cell permeant	Invitrogen (Waltham, MA)	Cat# R1245MP
RIPA Buffer	Sigma (St. Louis, MO)	Cat# R0278-50ml

Sodium phenylbutyrate (4PBA)	Sigma (St. Louis, MO)	Cat# SML0309
SuperSignal™ West Pico Plus ECL substrate	Thermo Fisher (Waltham, MA)	Cat# 34577
Tetramethylrhodamine, Ethyl Ester, Perchlorate (TMRE)	Thermo Fisher (Waltham, MA)	Cat# T669
Thiazolyl Blue Tetrazolium Bromide (MTT)	Sigma (St. Louis, MO)	Cat# M2128-500MG
TNF-α inhibitor	Enzo Life science (Farmingdale, NY)	Cat# ENZ-CHM119-0001
Trizol	Thermo Fisher (Waltham, MA)	Cat# 155596018
ZVAD-FMK	Enzo Life science (Farmingdale, NY)	Cat# ALX-260-020-M001
**Critical commercial kits and assays**		
ATPlite Luminescence Assay System	PerkinElmer (Waltham, MA)	Cat# 6016941
Comet Assay	Trevigen (Gaithersburg, MD)	Cat# 4250-050-K
Cytotoxicity Detection Kit PLUS (LDH)	Sigma (St. Louis, MO)	Cat# 4744926001
Direct-zol™ RNA MiniPrep	Zymo Research (Irvine, CA)	Cat# R2052
EnzChek™ Caspase-3 Assay Kit #2	Thermo Fisher (Waltham, MA)	Cat# E13184
Fast SYBR™ Green Master Mix	Thermo Fisher (Waltham, MA)	Cat# 4385612
Griess Reagent System	Promega (Madison, WI)	Cat# G2930
High-Capacity cDNA Reverse Transcription Kit	Thermo Fisher (Waltham, MA)	Cat# 4368813
Human IL-1β ELISA	BioLegend (San Diego, CA)	Cat# 437004
Human IL-6 DuoSet ELISA	R&D (Minneapolis, MN)	Cat# DY206-05
Human TNF-α DuoSet ELISA	R&D (Minneapolis, MN)	Cat# DY210-05
Mouse anti-dsDNA IgG-specific ELISA Kit	Alpha Diagnostic International (San Antonio, TX)	Cat# 5120
Mouse IL-6 DuoSet ELISA	R&D (Minneapolis, MN)	Cat# Dy-406-05
Mouse TNF-α DuoSet ELISA	R&D (Minneapolis, MN)	Cat# Dy-410-05
RC DC™ Protein Assay Kit	Bio-Rad (Hercules, CA)	Cat# 5000120
RNeasy Plus Mini kit	Qiagen (Germantown, MD)	Cat# 74134
TURBO DNA-free™ Kit	Invitrogen (Waltham, MA)	Cat# AM1907
**Software and Algorithms**		
Biorender	https://biorender.com/	
CFX Maestro	Bio-Rad (Hercules, CA)	Version 2.3 (5.3.022.1030)
DAVID bioinformatics database	https://david.ncifcrf.gov/	Version 6.8 [1]
Fiji Software	https://imagej.net/Fiji	Version 1.53c
Graph Pad Prism	https://www.graphpad.com/	Version 8.0
Heatmapper	http://www.heatmapper.ca	[2]
ImageJ	https://imagej.nih.gov/ij/	1.52a
iPathwayGuide	https://advaitabio.com/ipathwayguide/	Release January 31, 2020 [3]
MGI database	http://www.Informatics.jax.org/index.shtml	
**Deposited data**		
RNA-seq data	This paper and [4]	GEO accession numbers: GSE173877 GSE159821

**Other**		
BX41 Phase Contrast & Darkfield Microscope	Olympus	BX41
Nikon E800 Epifluorescence and Brightfield microscope	Nikon	E800
Real-Time PCR System	Bio-Rad (Hercules, CA)	CFX384 Touch
Real-Time PCR System	Thermo Fisher (Waltham, MA)	StepOnePlus

**List of primer sequences used for qRT-PCR analysis**		
**Gene Mouse**	**Forward**	**Reverse**
Apobec3	CAGCCATCGCAAATGCTATTC	ATTTCAGCGTGGATGTTGTCC
Cxcl10	GGATGGCTGTCCTAGCTCTG	TGAGCTAGGGAGGACAAGGA
Dnase1l3	TCTCGACTTGGAAGAAACACG	GTCTCCATCCTGATAGTCATGGT
Grp78	ACTTGGGGACCACCTATTCCT	ATCGCCAATCAGACGCTCC
Hprt	GCCCCAAAATGGTTAAGGTT	TTGCGCTCATCTTAGGCTTT
Ifi44	GGAGGATTTGCCTTTGAACA	TGGGTTAAAGGTCAGGGCTA
Ifi44L	ACAGGCTCATGAACCATCCA	TCTCGAGAACTCATGCTCCA
Ifitm1	GGAGCAGCAAGAGGTGGTTG	GATGTTCAGGCACTTGGCGG
Il-1b	CAGGCAGGCAGTATCACTCA	TGTCCTCATCCTGGAAGGTC
Irf-7	TGCTGTTTGGAGACTGGCTAT	TCCAAGCTCCCGGCTAAGT
Mx1-SNE1	CGGTTGTTTACCAAACTGCG	TTCCAGGGCTTTGACTCGC
Mxi1	GGCACACAACACTCGGTTTG	CCATTCGTATCCGCTCCATCT
Nos2	CACCTTGGAGTTCACCCAGT	ACCACTCGTACTTGGGATGC
Oas1a	GCCTGATCCCAGAATCTATGC	GAGCAACTCTAGGGCGTACTG
Polr2f	GACAACGAGGACAATTTCGACG	GGAGAATCTCGACATTTTCCTGG
Prkr	TGCCGTGGTTTTCCTTTAAC	CAGGCCCAGCAATTAACAAT
Rnaseh2a	GGATAGAGGTGACAGTCAAGGC	CCTGAGCCATAATCGGAGTCCA
Tnfa	CTGGGACAGTGACCTGGACT	CTCCCTTTGCAGAACTCAGG

Supplementary References

1 Huang, D. W., Sherman, B. T. & Lempicki, R. A. Systematic and integrative analysis of large gene lists using DAVID bioinformatics resources. *Nature Protocols* **4**, 44–57, doi:10.1038/nprot.2008.211 (2009).

2 Babicki, S. *et al.* Heatmapper: web-enabled heat mapping for all. *Nucleic Acids Res.* **44**, 147-153, doi:10.1093/nar/gkw419 (2016).

3 Ahsan, S. & Draghici, S. Identifying Significantly Impacted Pathways and Putative Mechanisms with iPathwayGuide. *Curr Protoc Bioinformatics*, 7.15.11–17.15.30, doi:10.1002/cpbi.24 (2017).

4 Drongelen, V. v. *et al.* HLA-DRB1 allelic epitopes that associate with autoimmune disease risk or protection activate reciprocal macrophage polarization. *Sci Rep* **11**, 1-15, doi:10.1038/s41598-021-82195-3 (2021).
