# Supplementary material for: The lupus susceptibility allele DRB1*03:01 encodes a disease-driving epitope
Source: Commun Biol. 2022 Jul 28;5:751. doi: 10.1038/s42003-022-03717-x (PMC9334592; doi:10.1038/s42003-022-03717-x)
Supplement: Supplementary file 2 — Description of Additional Supplementary Files [file 42003_2022_3717_MOESM2_ESM.pdf]

Description of Additional Supplementary Files

File Name: Supplementary Data 1

Description: RNA-seq data RAW 264.7 macrophages, Model A

File Name: Supplementary Data 2

Description: RNA-seq data RAW 264.7 macrophages, Model B

File Name: Supplementary Data 3
Description: RNA-seq data THP-1 macrophages, Model B

File Name: Supplementary Data 4

Description: The source data for figures 3-7 in the paper, and Supplementary figures 1-8.

.
