# Supplementary material for: The lupus susceptibility allele DRB1*03:01 encodes a disease-driving epitope
Source: Commun Biol. 2022 Jul 28;5:751. doi: 10.1038/s42003-022-03717-x (PMC9334592; doi:10.1038/s42003-022-03717-x)
Supplement: Supplementary file 7 — Reporting Summary [file 42003_2022_3717_MOESM7_ESM.pdf]

nature portfolio

Corresponding author(s): Joseph Holoshitz
Last updated by author(s): Jun 7, 2022

Reporting Summary

Nature Portfolio wishes to improve the reproducibility of the work that we publish. This form provides structure for consistency and transparency in reporting. For further information on Nature Portfolio policies, see our Editorial Policies and the Editorial Policy Checklist.

Statistics

For all statistical analyses, confirm that the following items are present in the figure legend, table legend, main text, or Methods section.

n/a	Confirmed	
☐	☒	The exact sample size (n) for each experimental group/condition, given as a discrete number and unit of measurement
☐	☒	A statement on whether measurements were taken from distinct samples or whether the same sample was measured repeatedly
☐	☒	The statistical test(s) used AND whether they are one- or two-sided<br>*Only common tests should be described solely by name; describe more complex techniques in the Methods section.*
☒	☐	A description of all covariates tested
☒	☐	A description of any assumptions or corrections, such as tests of normality and adjustment for multiple comparisons
☐	☒	A full description of the statistical parameters including central tendency (e.g. means) or other basic estimates (e.g. regression coefficient) AND variation (e.g. standard deviation) or associated estimates of uncertainty (e.g. confidence intervals)
☐	☒	For null hypothesis testing, the test statistic (e.g. F, t, r) with confidence intervals, effect sizes, degrees of freedom and P value noted<br>*Give P values as exact values whenever suitable.*
☒	☐	For Bayesian analysis, information on the choice of priors and Markov chain Monte Carlo settings
☒	☐	For hierarchical and complex designs, identification of the appropriate level for tests and full reporting of outcomes
☒	☐	Estimates of effect sizes (e.g. Cohen's d, Pearson's r), indicating how they were calculated

Our web collection on statistics for biologists contains articles on many of the points above.

Software and code

Policy information about availability of computer code

Data collection	qRT-PCR data were acquired using a StepOnePlus or a CFX384 Touch (Bio-Rad) Real-Time PCR system.<br>Western Blot images were acquired using a Omega Lum C imaging system or ChemiDoc™ Imaging System (Bio-rad).<br>Absorbance, luminescence and fluorescence data were acquired using a Synergy H1 Hybrid Multi-Mode Reader.<br>Immunofluorescence images were taken using a Nikon E800 Epifluorescence microscope (Nikon).<br>Brightfield images were taken with a BX41 microscope and an DP73 camera (Olympus).<br>RNA-seq for was acquired using Illumina's HiSeq2500v4.
Data analysis	Graphs and statistical analysis were generated using GraphPad Prism (version 8.0).<br>qRT-PCR data was analyzed using StepOne (2.3) or CFX Maestro (version 2.3) software.<br>Western blot images were analyzed using Image J.<br>RNA-seq data was analyzed using R-3.4.1, DESeq2-1.16.1, featureCounts, Rsubread1.5.0p3 package (RAW264.7 cells), Rsubread-1.6.1 package (THP-1 cells), DAVID, iPathwayGuide (Advaita), heatmapper.

For manuscripts utilizing custom algorithms or software that are central to the research but not yet described in published literature, software must be made available to editors and reviewers. We strongly encourage code deposition in a community repository (e.g. GitHub). See the Nature Portfolio guidelines for submitting code & software for further information.

Data

Policy information about availability of data

All manuscripts must include a data availability statement. This statement should provide the following information, where applicable:

- Accession codes, unique identifiers, or web links for publicly available datasets
- A description of any restrictions on data availability
- For clinical datasets or third party data, please ensure that the statement adheres to our policy

RNA-seq datasets generated in this manuscript were deposited in the NCBI Gene Expression Omnibus and are accessible through GEO accession number GSE173877. Source data are provided with this paper. Additional data supporting the findings of this study are available from the Lead Contact upon request.

Field-specific reporting

Please select the one below that is the best fit for your research. If you are not sure, read the appropriate sections before making your selection.

☒ Life sciences ☐ Behavioural & social sciences ☐ Ecological, evolutionary & environmental sciences

For a reference copy of the document with all sections, see nature.com/documents/nr-reporting-summary-flat.pdf

Life sciences study design

All studies must disclose on these points even when the disclosure is negative.

Sample size	Sample size was determined based on earlier experience, pilot experiments or literature. Animal experiments were performed three times using 5-7 animals in each experiment.
Data exclusions	Outliers were excluded from data analysis. Basis for data exclusion was based on a Z-score of over 3 or additional experiments/additional data aquired.
Replication	All experiments were repeated multiple times as stated in the text, figure legends and methods.
Randomization	For in vivo and ex vivo animal studies, animals were age and sex matched and were selected randomly into treatment groups.
Blinding	For pathological scoring, the pathologist was blinded to both mouse strain and treatment. For immunofluorescence scoring, the interpreter was blinded to mouse strain and treatment.

Reporting for specific materials, systems and methods

We require information from authors about some types of materials, experimental systems and methods used in many studies. Here, indicate whether each material, system or method listed is relevant to your study. If you are not sure if a list item applies to your research, read the appropriate section before selecting a response.

Materials & experimental systems

n/a	Involved in the study	
☐	☒	Antibodies
☐	☒	Eukaryotic cell lines
☒	☐	Palaeontology and archaeology
☐	☒	Animals and other organisms
☒	☐	Human research participants
☒	☐	Clinical data
☒	☐	Dual use research of concern

Methods

n/a	Involved in the study	
☒	☐	ChIP-seq
☒	☐	Flow cytometry
☒	☐	MRI-based neuroimaging

Antibodies

Antibodies used

Anti-C3 (ICL, #GC3-90F-Z)
Anti-Mouse CHOP (CST, # 2895)
Anti-Rabbit IRE1α (Novus Biologicals, #NB100-2323SS)
Anti-Mouse BiP/Grp78 (BD Biosciences, #610979)
Anti-Mouse β-actin (BD Biosciences, #612657)
Anti-mouse Mono- and polyubiquitinylated conjugates (FK2, Enzo Life science, #BML-PW8810)
Mouse Anti-RIP (BD Biosciences, #610458)
Anti-Rabbit Recombinant Anti-MLKL (pS345, Abcam, #ab196436)
Anti-Rabbit SQSTM1/p62 (CST, #5114S)

	Anti-Rabbit LC3B (CST, Cat# 2775S)<br>Anti-rabbit IgG, HRP linked (CST, #7074S)<br>Anti-mouse IgG HRP-linked (GE healthcare Lifesciences, #NA931)<br>Goat anti-Mouse IgG (H+L) Alexa Fluor 647 conjungated (Thermo Scientific, #A-21235)<br>Beclin-1 Antibody (CST, # 3738)<br>IFNAR2 Monoclonal Antibody (MMHAR-2) (PBL Assay Science, # 21385-1)
Validation	All antibodies have been validated by the manufacturer (data available on manufacturers website).<br>Anti-β-Actin has been validated by others (publications available on manufacturers website).<br>Anti-mouse IgG HRP-conjugated has been used and validated by us earlier (e.g. Fu et al., Proc Natl Acad Sci U S A. 2018).

Eukaryotic cell lines

Policy information about cell lines

Cell line source(s)	RAW 264.7 (ATCC); THP-1 (ATCC)
Authentication	Authenticated by vendor
Mycoplasma contamination	Cell lines were tested negative for Mycoplasma contamination
Commonly misidentified lines<br>(See ICLAC register)	The cell lines used for this study do not belong to the group of commonly misidentified cell lines according to the ICLAC Register of Misidentified Cell Lines (v11).

Animals and other organisms

Policy information about studies involving animals; ARRIVE guidelines recommended for reporting animal research

Laboratory animals	Transgenic mice LE-Tg, SE-Tg and PE-Tg, female and male were used. In vivo experiments started at 8-10 week of age and lasted for 24 weeks. Additionally, 3 genetic background control WT mouse strains, B6J, B10J and SWR were used.
Wild animals	This study did not involve wild animals.
Field-collected samples	This study did not involve samples collected from the field.
Ethics oversight	All animal experiments were performed in accordance with by the University of Michigan Unit for Laboratory Animal Medicine and by the University of Michigan Committee on Use and Care of Animals using approved protocols and in accordance with federal, state, local, and institutional laws, regulations, policies, and standards for animal research.

Note that full information on the approval of the study protocol must also be provided in the manuscript.
